# An Early Longitudinal Decrease in Platelet Count Is Associated With Poor Prognosis in Acute Myocardial Infarction Patients: A Multicenter Retrospective Study

**DOI:** 10.1002/clc.70504

**Published:** 2026-10-22

**Authors:** Tianlai Lin, Jingqing Xu, Xiaoling Zeng, Qiang Liu, Mouying Liu, Xizhe Chen, Youli Chen

**Affiliations:** ^1^ Department of Intensive Care Unit Quanzhou First Hospital Affiliated to Fujian Medical University Fujian China; ^2^ Fujian Provincial Key Laboratory of Difficult and Critical Disease Research; Department of Critical Care Medicine Fuzhou University Affiliated Provincial Hospital Fujian China

**Keywords:** acute myocardial infarction, mortality, platelet

## Abstract

**Background:**

Limited data are available on the association between early longitudinal changes in platelet count and mortality.

**Objective:**

This study aimed to compare the early longitudinal changes in platelet count between survivors and non‐survivors.

**Methods:**

This was a retrospective multi‐center cohort study using data from the eICU Collaborative Research Database. A total of 6,769 patients diagnosed with AMI were included in the study. The analysis employed a Generalized Additive Model (GAM) and a Generalized Additive Mixed Model (GAMM) to compare longitudinal changes in platelet count between survivors and non‐survivors.

**Results:**

The ICU 28‐day and ICU 7‐day all‐cause mortality were 6.1% and 4.6%, respectively. During the first 5 days of ICU admission, GAM showed that non‐survivors exhibited a significantly greater decline in platelet count than survivors. Specifically, the GAMM results showed that the difference in platelet count between ICU 28‐day non‐survivors and ICU 28‐day survivors increased by an average of 15.4 × 10^9^/L daily (95% Confidence Interval [CI], 14.1 to 16.7; *p* < 0.001). Similarly, the difference in platelet count between ICU 7‐day non‐survivors and ICU 7‐day survivors increased by an average of 18.0 × 10^9^/L daily (95% CI, 16.1−19.8; *p* < 0.001).

**Conclusions:**

An early longitudinal decline in platelet count was associated with a poor prognosis in patients with AMI. However, this study is observational, and causality cannot be inferred.

## Graphical abstract

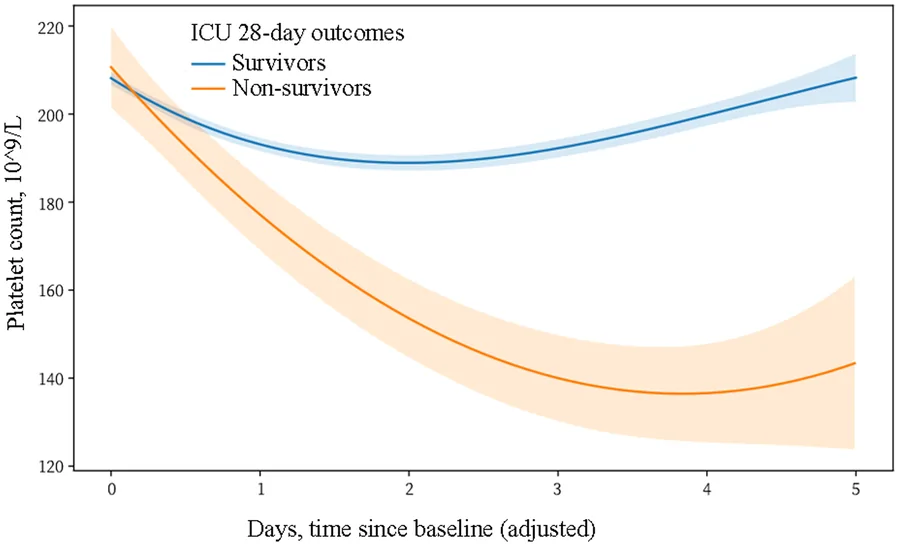

**Abbreviations**
AMI: acute myocardial infarction
CI: confidence interval
CITI: Collaborative Institutional Training Initiative
eICU‐CRD: eICU Collaborative Research Database
GAM: Generalized Additive Models
GAMM: Generalized Additive Mixed Models
GEE: Generalized Estimating Equations
HIPAA: Health Insurance Portability and Accountability Act
HIT: Heparin‐induced thrombocytopenia
ICD: international classification of diseases
ICU: intensive care unit
IQR: interquartile range
NSTEMI: non‐ST‐segment elevation myocardial infarction
PTCA: percutaneous transluminal coronary angioplasty
SD: standard deviation
SOFA: sequential organ failure assessment
STEMI: ST‐segment elevation myocardial infarction
VIF: variance inflation factor

## 1 Introduction

Acute myocardial infarction (AMI) is characterized by myocardial necrosis resulting from unstable ischemic syndrome [1]. As a leading cause of morbidity and mortality worldwide, AMI places a significant burden on healthcare systems [2]. Therefore, early identification of critically ill patients is crucial for improving prognosis [3].

Platelets may play a vital role in the pathophysiology of AMI [4, 5]. Platelets adhere to the vulnerable coronary plaque, which may initiate thrombotic occlusion and result in myocardial ischemia and infarction [5]. Many studies have assessed the relationship between platelet counts on admission and mortality in AMI patients [6, 7, 8, 9, 10, 11, 12, 13]. However, the prognostic value of a single baseline platelet count remains controversial, as mortality has been inconsistently linked to both low and high platelet levels. This may be partly because a single platelet count may only reflect the disease state at a specific point in time and cannot capture the dynamic changes in disease progression [14, 15, 16].

Only a few studies have described the changes in platelet count over time in patients with AMI [6, 17]. A previous study showed that platelet counts in patients with AMI gradually decrease after admission and rebound 1 week later [6]. Furthermore, large‐scale epidemiological data indicated that the development of thrombocytopenia during AMI significantly increases the risk of severe complications and nearly doubles in‐hospital mortality [6]. Platelet counts may fluctuate due to ongoing platelet consumption, systemic inflammation, and antithrombotic therapies. A rapid decline or failure to recover platelet levels may be associated with multiorgan dysfunction and a higher risk of mortality [3, 18]. To the best of our knowledge, assessments of the association between early longitudinal platelet count and mortality in patients with AMI have not been reported. Therefore, this study aimed to explore the early dynamic changes in platelet count over time between AMI survivors and non‐survivors.

## 2 Materials and Methods

### 2.1 Data Source

The eICU Collaborative Research Database (eICU‐CRD), developed by Philips Healthcare, is a publicly accessible multi‐center database [19]. It contains de‐identified data from over 200 000 ICU admissions at 208 ICU hospitals in the United States between 2014 and 2015 [19]. According to the data usage agreement of the PhysioNet Review Board, access to the database is possible by passing an examination and obtaining certification. Author Youli Chen was granted access to the eICU‐CRD data (Certification ID: 66408921) after completing the Collaborative Institutional Training Initiative (CITI) program “Data or Specimens Only Research.” The requirement for ethical approval and patient informed consent was waived because this study was retrospective, there was no direct patient intervention, and the database was released under the Health Insurance Portability and Accountability Act (HIPAA) safe harbor provision (HIPAA Certification No. 1031219‐2). The study conformed to the principles outlined in the Declaration of Helsinki.

### 2.2 Study Population

This study included 9821 patients diagnosed with AMI who were assigned an International Classification of Diseases (ICD)‐9 code of 410.x. The exclusion criteria were: (1) 1273 patients missing baseline platelet count within 24 h after ICU admission; (2) 1725 patients with only one recorded platelet count after ICU admission; (3) 45 patients with platelet concentrate transfusion; (4) 9 patients with heparin‐induced thrombocytopenia (HIT). Finally, a total of 6769 patients with AMI were included in this study. Among the included cohort, 3841 (56.7%) patients were diagnosed with non‐ST‐segment elevation myocardial infarction (NSTEMI) and 2928 (43.3%) with ST‐segment elevation myocardial infarction (STEMI). The study flowchart is presented in Figure 1.

**Figure 1:**
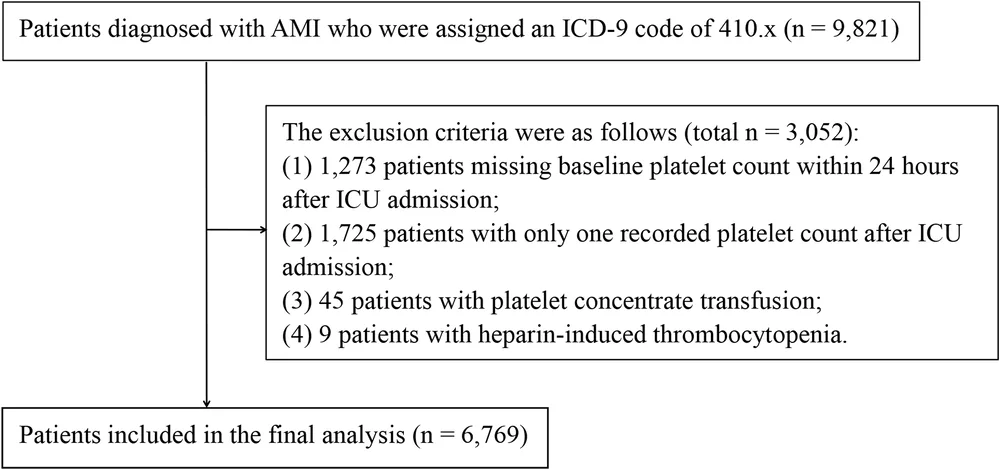
Flow diagram of the study.

### 2.3 Variables

Clinical and demographic data were extracted from the eICU‐CRD, including age, gender, ethnicity, body mass index, temperature, mean arterial pressure, Glasgow Coma Scale score, Sequential Organ Failure Assessment (SOFA), and Acute Physiology Score. Comorbidities and complications included diabetes mellitus, chronic heart failure, pneumonia, sepsis, stroke, bleeding, hemorrhage, and HIT. The initial laboratory variables extracted within the first 24 h of ICU admission encompassed blood urea nitrogen, serum creatinine, red blood cell count, hemoglobin, white blood cell count, platelet count, bilirubin, and lactate. Other clinical data included microbiological cultures, vasoactive agents (dobutamine, dopamine, epinephrine, norepinephrine), medications (aspirin, clopidogrel, heparin, enoxaparin, warfarin), and specific treatments (platelet concentrate transfusion, thrombolytic therapy, mechanical ventilation, and percutaneous transluminal coronary angioplasty [PTCA]). Details of the data extraction are summarized in Table S1.

The SOFA score was calculated using the worst values recorded within the first 24 h following ICU admission. Consistent with prior studies [20, 21], missing organ components were assigned a score of 0, and patients with documented vasopressor use but missing dose data [µg/(kg·min)] were categorized into the lowest dose tier. Ages recorded as “>89” in the database were capped at 89 years for data de‐identification.

### 2.4 Outcomes

The primary outcome was all‐cause ICU 28‐day mortality. The secondary outcome was all‐cause ICU 7‐day mortality. Patients who were discharged or transferred alive from the ICU before reaching the 7‐ or 28‐day endpoints were considered survivors at these respective time points.

### 2.5 Statistical Analysis

Continuous variables with a normal distribution were presented as mean ± standard deviation (SD) and analyzed using the *t*‐test. Continuous variables with a skewed distribution were presented as median (interquartile range [IQR]) and analyzed using the Kruskal−Wallis test. Categorical variables were presented as *n* (%) and analyzed using the chi‐square test.

For longitudinal analysis, platelet counts were assessed over time. The initial measurement was defined as baseline (time = 0), and subsequent measurements were recorded as the exact elapsed time (in days) from the baseline. Generalized Additive Models (GAM) and Generalized Additive Mixed Models (GAMM) are exceptionally well‐suited for analyzing repeated‐measures data, particularly when dealing with irregular measurement intervals [3, 22]. GAM was used to visualize the non‐linear association of platelet counts over time for both survivors and non‐survivors. GAMM was constructed to test the longitudinal differences between the two groups. Time was treated as a continuous variable with a time‐by‐mortality interaction term, and intra‐subject correlation was adjusted for via random intercepts. Multicollinearity was assessed using the variance inflation factor (VIF). Dummy variables were used to indicate missing covariate values. The code specifying spline bases, knot placements, penalization, and model diagnostics is provided in the Supporting Information Material.

To explore the relationship between changes in platelet count over time (during the first 5 days) and patient mortality in AMI patients, sensitivity analyses were conducted: (1) employing a Generalized Estimating Equations (GEE) model to account for within‐patient correlation arising from repeated ICU admissions; (2) using GAM analysis within the following patient subgroups: (1) patients with three or more platelet measurements; (2) patients who did not receive vasopressors within the first 24 h of ICU admission; (3) patients with NSTEMI; (4) patients with STEMI; (5) patients aged < 65 years; (6) patients aged ≥ 65 years; (7) propensity score‐matched cohort. Propensity score matching was employed to identify patients with similar baseline characteristics, addressing the observed differences in baseline characteristics between ICU 28‐ and 7‐day non‐survivors and survivors. Matching was performed using a 1:1 protocol with a greedy matching algorithm without replacement, and a caliper width equal to 0.05 of the SD of the logit of the propensity score.

All statistical analyses were performed using EmpowerStats (www.empowerstats.com) and R software (http://www.R-project.org, X&Y Solutions Inc., Boston, MA, USA). A two‐tailed *p* value of <0.05 was considered statistically significant.

## 3 Results

Platelet counts for patients with AMI at different time points were summarized in Table 1. On ICU admission, the median and mean platelet counts for all patients were 202.0 (IQR, 158.0−252.0) ×10^9^/L and 211.7 (SD, 84.8) ×10^9^/L, respectively. From Day 2 through Day 7, platelet counts were consistently and significantly lower in non‐survivors than in survivors across both ICU 28‐day and ICU 7‐day mortality cohorts (all *p* < 0.05).

**Table 1:** Platelet count differences between survivors and non‐survivors.

Time	Platelet count, 10^9^/L, median (Q1 ‐ Q3)					
	On ICU admission	Days 2−3	Days 3−4	Days 4−5	Days 6−7	Days 14−15
*ICU 28‐day mortality*						
Survivors, *n* = 6359	202.0 (158.0‐251.0)	176.0 (130.0‐230.0)	181.0 (130.0‐243.0)	189.0 (137.0‐249.5)	210.0 (150.0‐281.0)	266.0 (187.0‐383.0)
Non‐survivors, *n* = 410	202.0 (148.2‐265.0)	132.5 (84.8‐197.0)	129.5 (77.0‐197.8)	128.0 (76.0‐194.0)	125.5 (78.2‐201.5)	221.0 (157.0‐364.8)
*p* value	0.958	< 0.001	< 0.001	< 0.001	< 0.001	0.244
*ICU 7‐day mortality*						
Survivors, *n* = 6457	202.0 (158.0‐251.0)	175.0 (128.0‐230.0)	180.0 (128.0‐241.0)	187.0 (134.0‐248.0)	206.0 (143.0‐278.0)	—
Non‐survivors, *n* = 312	198.5 (143.8‐269.0)	135.5 (81.5‐186.5)	132.5 (73.8‐195.0)	124.5 (84.5‐199.0)	153.0 (89.2‐174.0)	—
*p* value	0.900	<0.001	<0.001	<0.001	0.027	—
Total	202.0 (158.0‐252.0)	174.0 (126.0‐229.0)	179.0 (126.0‐240.0)	186.0 (133.0‐247.2)	206.0 (143.0‐278.0)	264.5 (184.0‐382.8)

Table 2 presents the baseline characteristics of the 6,769 patients with AMI. The ICU 28‐day mortality was 6.1% (410/6,769), and the ICU 7‐day mortality was 4.6% (312/6,769). Compared with survivors, non‐survivors had the following characteristics: (1) older age and greater disease severity on admission (characterized by lower mean arterial pressure, higher SOFA and Acute Physiology Score, and a higher proportion of Glasgow Coma Scale score ≤ 8); (2) higher rates of comorbidities and complications (included chronic heart failure, pneumonia, sepsis, stroke, and bleeding events); (3) worse laboratory values (accompanied by impaired renal function, higher white blood cell counts, and elevated lactate levels); and (4) a greater requirement for intensive clinical interventions (such as higher use of mechanical ventilation and vasoactive agents). There were no significant differences between the two groups in the rates of PTCA, thrombolytic therapy, or heparin use.

**Table 2:** Baseline characteristics and clinical outcomes of acute myocardial infarction patients.

Characteristics	Total	ICU 28‐day mortality			ICU 7‐day mortality		
		Survivors	Non‐survivors	*p* value	Survivors	Non‐survivors	*p* value
*N*	6769	6359	410		6457	312	
Age, years	67.0 (57.0‐77.0)	67.0 (57.0‐77.0)	73.0 (65.0‐81.0)	< 0.001	67.0 (57.0‐77.0)	74.0 (66.0‐83.0)	< 0.001
*Gender*				0.082			0.405
Female	2518 (37.2%)	2349 (36.9%)	169 (41.2%)		2395 (37.1%)	123 (39.4%)	
Male	4,251 (62.8%)	4,010 (63.1%)	241 (58.8%)		4,062 (62.9%)	189 (60.6%)	
*Ethnicity*				0.011			0.016
Caucasian	5243 (77.5%)	4903 (77.1%)	340 (82.9%)		4,983 (77.2%)	260 (83.3%)	
African American	628 (9.3%)	605 (9.5%)	23 (5.6%)		612 (9.5%)	16 (5.1%)	
Other/unknown	898 (13.3%)	851 (13.4%)	47 (11.5%)		862 (13.3%)	36 (11.5%)	
Body mass index, kg/m^2^	29.4 ± 7.7	29.4 ± 7.5	29.2 ± 10.8	0.520	29.4 ± 7.5	29.0 ± 11.0	0.347
Temperature,°C	36.5 (36.2‐36.7)	36.5 (36.2‐36.7)	36.2 (34.9‐36.6)	< 0.001	36.5 (36.2‐36.7)	36.2 (34.8‐36.6)	< 0.001
Mean arterial pressure, mmHg	64.0 (53.0‐121.0)	65.0 (53.0‐121.0)	55.0 (45.0‐128.0)	< 0.001	65.0 (53.0‐121.0)	54.0 (45.0‐127.5)	< 0.001
*Glasgow coma scale score*				< 0.001			< 0.001
≤8	681 (11.0%)	524 (9.0%)	157 (42.5%)		562 (9.5%)	119 (41.6%)	
>8	5494 (89.0%)	5282 (91.0%)	212 (57.5%)		5327 (90.5%)	167 (58.4%)	
SOFA	2.0 (1.0‐5.0)	2.0 (1.0‐4.0)	7.0 (4.0‐10.0)	< 0.001	2.0 (1.0‐4.0)	7.0 (4.0‐10.0)	< 0.001
Acute physiology score	36.0 (26.0‐52.8)	35.0 (25.0‐49.0)	76.0 (54.0‐103.0)	< 0.001	35.0 (25.0‐50.0)	76.5 (54.0‐104.0)	< 0.001
Diabetes mellitus	1100 (16.3%)	1026 (16.1%)	74 (18.0%)	0.309	1043 (16.2%)	57 (18.3%)	0.322
Chronic heart failure	1045 (15.4%)	942 (14.8%)	103 (25.1%)	< 0.001	970 (15.0%)	75 (24.0%)	< 0.001
Pneumonia	761 (11.2%)	658 (10.3%)	103 (25.1%)	< 0.001	694 (10.7%)	67 (21.5%)	< 0.001
Sepsis	747 (11.0%)	634 (10.0%)	113 (27.6%)	< 0.001	666 (10.3%)	81 (26.0%)	< 0.001
Stroke	211 (3.1%)	182 (2.9%)	29 (7.1%)	< 0.001	191 (3.0%)	20 (6.4%)	< 0.001
Bleeding	713 (10.5%)	637 (10.0%)	76 (18.5%)	< 0.001	659 (10.2%)	54 (17.3%)	< 0.001
Hemorrhage	66 (1.0%)	57 (0.9%)	9 (2.2%)	0.009	60 (0.9%)	6 (1.9%)	0.081
Blood urea nitrogen, mg/dL	19.0 (14.0‐31.0)	19.0 (13.0‐30.0)	32.0 (20.0‐50.0)	< 0.001	19.0 (13.0‐30.0)	32.0 (20.0‐49.8)	< 0.001
Serum creatinine, mg/dL	1.0 (0.8‐1.5)	1.0 (0.8‐1.5)	1.7 (1.1‐2.5)	< 0.001	1.0 (0.8‐1.5)	1.7 (1.1‐2.5)	< 0.001
Red blood cell count, 10^12^/L	4.0 ± 0.8	4.0 ± 0.8	3.8 ± 0.8	< 0.001	4.0 ± 0.8	3.8 ± 0.8	0.001
Hemoglobin, g/dL	12.0 (10.1‐13.6)	12.0 (10.2‐13.7)	11.4 (9.7‐13.1)	< 0.001	12.0 (10.2‐13.6)	11.4 (9.8‐13.4)	0.004
White blood cell count, 10^9^/L	11.3 (8.7‐14.8)	11.1 (8.6‐14.5)	14.8 (11.1‐20.6)	< 0.001	11.2 (8.6‐14.6)	15.2 (11.3‐21.7)	< 0.001
Lactate, mmol/L	1.9 (1.3‐3.5)	1.8 (1.2‐3.1)	3.8 (1.9‐6.7)	< 0.001	1.8 (1.2‐3.2)	4.0 (2.0‐6.9)	< 0.001
Microbiological culture	129 (1.9%)	114 (1.8%)	15 (3.7%)	0.007	116 (1.8%)	13 (4.2%)	0.003
Dobutamine	151 (2.2%)	125 (2.0%)	26 (6.3%)	< 0.001	131 (2.0%)	20 (6.4%)	< 0.001
*Dopamine*				< 0.001			< 0.001
No	6,476 (95.7%)	6,107 (96.0%)	369 (90.0%)		6,202 (96.1%)	274 (87.8%)	
≤5, µg/(kg·min)	48 (0.7%)	41 (0.6%)	7 (1.7%)		41 (0.6%)	7 (2.2%)	
>5, µg/(kg·min)	36 (0.5%)	33 (0.5%)	3 (0.7%)		34 (0.5%)	2 (0.6%)	
>15, µg/(kg·min)	15 (0.2%)	9 (0.1%)	6 (1.5%)		9 (0.1%)	6 (1.9%)	
unknown dose	194 (2.9%)	169 (2.7%)	25 (6.1%)		171 (2.6%)	23 (7.4%)	
*Epinephrine*				< 0.001			< 0.001
no	6,632 (98.0%)	6,264 (98.5%)	368 (89.8%)		6,357 (98.5%)	275 (88.1%)	
≤0.1, µg/(kg·min)	3 (0.0%)	2 (0.0%)	1 (0.2%)		3 (0.0%)	0 (0.0%)	
>0.1, µg/(kg·min)	3 (0.0%)	2 (0.0%)	1 (0.2%)		2 (0.0%)	1 (0.3%)	
Unknown dose	131 (1.9%)	91 (1.4%)	40 (9.8%)		95 (1.5%)	36 (11.5%)	
*Norepinephrine*				< 0.001			< 0.001
No	6022 (89.0%)	5772 (90.8%)	250 (61.0%)		5835 (90.4%)	187 (59.9%)	
≤0.1, µg/(kg·min)	34 (0.5%)	29 (0.5%)	5 (1.2%)		30 (0.5%)	4 (1.3%)	
>0.1, µg/(kg·min)	44 (0.7%)	27 (0.4%)	17 (4.1%)		32 (0.5%)	12 (3.8%)	
unknown dose	669 (9.9%)	531 (8.4%)	138 (33.7%)		560 (8.7%)	109 (34.9%)	
Aspirin	3859 (57.0%)	3660 (57.6%)	199 (48.5%)	< 0.001	3716 (57.5%)	143 (45.8%)	< 0.001
Clopidogrel	1395 (20.6%)	1337 (21.0%)	58 (14.1%)	< 0.001	1352 (20.9%)	43 (13.8%)	0.002
Heparin	2794 (41.3%)	2616 (41.1%)	178 (43.4%)	0.364	2666 (41.3%)	128 (41.0%)	0.927
Enoxaparin	882 (13.0%)	842 (13.2%)	40 (9.8%)	0.042	858 (13.3%)	24 (7.7%)	0.004
Warfarin	389 (5.7%)	381 (6.0%)	8 (2.0%)	< 0.001	384 (5.9%)	5 (1.6%)	0.001
Thrombolytic therapy	54 (0.8%)	49 (0.8%)	5 (1.2%)	0.322	51 (0.8%)	3 (1.0%)	0.739
Mechanical ventilation	1743 (25.7%)	1496 (23.5%)	247 (60.2%)	< 0.001	1555 (24.1%)	188 (60.3%)	< 0.001
PTCA	2864 (42.3%)	2703 (42.5%)	161 (39.3%)	0.198	2730 (42.3%)	134 (42.9%)	0.815
ICU length of stay, days	2.1 (1.2‐3.9)	2.0 (1.2‐3.8)	3.4 (1.8‐6.8)	< 0.001	2.1 (1.2‐3.9)	2.7 (1.6‐3.9)	0.010

*Note:* The number of patients with missing data for each variable was as follows: lactate (*n* = 5257), Acute Physiology Score III (*n* = 1,091), temperature (*n* = 820), Glasgow Coma Scale score (*n* = 594), mean arterial pressure (*n* = 525), body mass index (*n* = 279), blood urea nitrogen (*n* = 195), serum creatinine (*n* = 184), red blood cell count (*n* = 53), white blood cell count (*n* = 49), and hemoglobin (*n* = 22).

Abbreviations: ICU, intensive care unit; PTCA, percutaneous transluminal coronary angioplasty; SOFA, Sequential Organ Failure Assessment.

After excluding hemoglobin (which exhibited a VIF of 5.2), all VIF values for all remaining variables were below the predefined threshold of 5. These variables were retained in the models (Table S2).

The GAM showed the early longitudinal changes in platelet count over the first 5 days for both ICU 28‐day and ICU 7‐day survivors and non‐survivors (Figure 2). Specifically, platelet counts were consistently and significantly lower in non‐survivors than in survivors across both cohorts. Sensitivity analyses restricted to other early time windows (the first 3 and 7 days) further confirmed the robustness of this early disparity (Figure S1). Similarly, GAM revealed longitudinal changes in platelet count over the first 28 days for ICU 28‐day survivors and non‐survivors (Figure S2). Specifically, both groups exhibited an initial decline in platelet count, followed by a subsequent increase.

**Figure 2:**
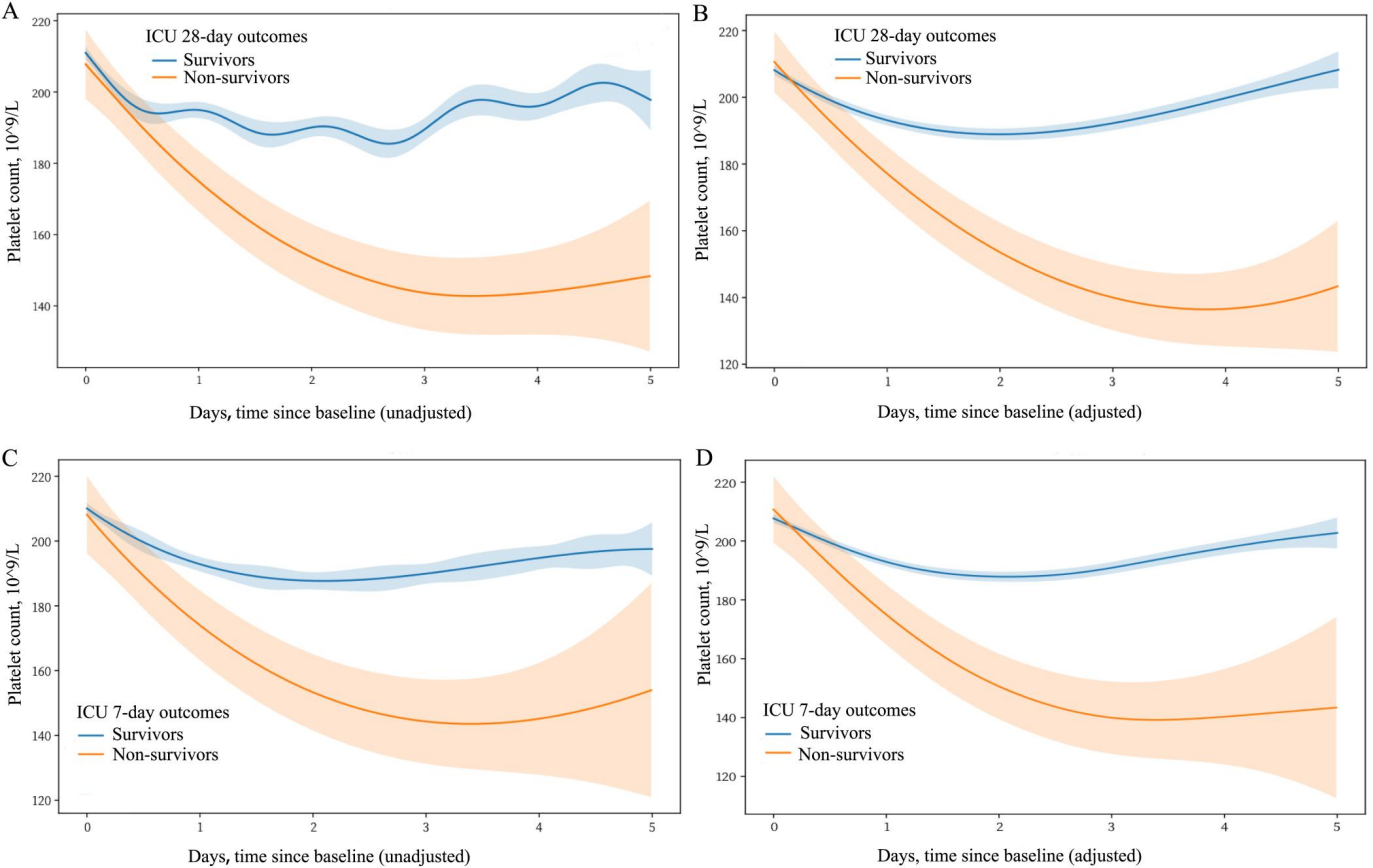
Changes in platelet count over time (the first 5 days) in AMI non‐survivors and survivors. (A) ICU 28‐day outcome: unadjusted model; (B) ICU 28‐day outcome: adjusted model; (C) ICU 7‐day outcome: unadjusted model; (D) ICU 7‐day outcome: adjusted model. Adjustment variables include: age, gender, ethnicity, body mass index, temperature, mean arterial pressure, Glasgow Coma Scale score, SOFA, Acute Physiology Score III, diabetes mellitus, chronic heart failure, pneumonia, sepsis, stroke, bleeding, hemorrhage, blood urea nitrogen, serum creatinine, red blood cell count, white blood cell count, lactate, microbiological culture, dobutamine, dopamine, epinephrine, norepinephrine, aspirin, clopidogrel, heparin, enoxaparin, warfarin, thrombolytic therapy, mechanical ventilation, and PTCA.

Table 3 presents the association between early longitudinal changes (the first 5 days) in platelet count and mortality among patients with AMI. After adjusting for potential confounders, the difference in platelet count between ICU 28‐day non‐survivors and ICU 28‐day survivors increased by an average of 15.4 × 10^9^/L per day (95% confidence interval [CI], 14.1 to 16.7; *p* < 0.001). Similarly, for ICU 7‐day mortality, the difference in platelet count between ICU 7‐day non‐survivors and ICU 7‐day survivors increased by an average of 18.0 × 10^9^/L per day (95% CI, 16.1 to 19.8; *p* < 0.001).

**Table 3:** Early changes in platelet counts over time (within the first 5 days) among patients with acute myocardial infarction.

Outcome	Unadjusted		Model Ⅰ		Model Ⅱ	
	*β* (95% CI)	*p* value	*β* (95% CI)	*p* value	β (95% CI)	*p* value
*ICU 28‐day mortality*						
Intercept	205.2 (203.2, 207.2)	< 0.001	247.8 (232.2, 263.3)	< 0.001	191.1 (109.1, 273.2)	< 0.001
Day	−1.6 (−1.9, −1.3)	< 0.001	−1.6 (−1.9, −1.3)	< 0.001	−1.6 (−1.9, −1.2)	< 0.001
Death	−1.9 (−10.0, 6.3)	0.653	7.4 (−0.9, 15.7)	0.079	19.2 (11.0, 27.5)	< 0.001
Day × death	−15.2 (−16.5, −13.9)	< 0.001	−15.2 (−16.5, −13.9)	< 0.001	−15.4 (−16.7, −14.1)	< 0.001
*ICU 7‐day mortality*						
Intercept	205.4 (203.4, 207.4)	< 0.001	247.2 (231.7, 262.8)	< 0.001	190.9 (108.8, 273.0)	< 0.001
Day	−2.1 (−2.4, −1.7)	< 0.001	−2.0 (−2.4, −1.7)	< 0.001	−2.0 (−2.3, −1.7)	< 0.001
Death	−1.2 (−10.5, 8.1)	0.799	9.2 (−0.2, 18.6)	0.056	22.0 (12.8, 31.3)	< 0.001
Day × death	−17.7 (−19.6, −15.9)	< 0.001	−17.8 (−19.6, −16.0)	< 0.001	−18.0 (−19.8, −16.1)	< 0.001

*Note:* Intercept: the mean platelet count at baseline (day = 0) for the survivor group (death = 0). Day: the mean daily change in platelet count over time for the survivor group (death = 0). Death: the difference in baseline platelet count (at day = 0) between the non‐survivor (death = 1) and survivor (death = 0) groups. Day × death: the difference in the mean daily change in platelet count between the non‐survivor (death = 1) and survivor (death = 0) groups. Model I: adjusted for age, gender, ethnicity, body mass index, and Glasgow Coma Scale score. Model II: adjusted for age, gender, ethnicity, body mass index, temperature, mean arterial pressure, Glasgow Coma Scale score, SOFA, Acute Physiology Score III, diabetes mellitus, chronic heart failure, pneumonia, sepsis, stroke, bleeding, hemorrhage, blood urea nitrogen, serum creatinine, red blood cell count, white blood cell count, lactate, microbiological culture, dobutamine, dopamine, epinephrine, norepinephrine, aspirin, clopidogrel, heparin, enoxaparin, warfarin, thrombolytic therapy, mechanical ventilation, PTCA.

Abbreviation: CI, confidence interval.

Sensitivity analyses were conducted: (1) GEE analysis revealed a greater decline in platelet count among non‐survivors compared to survivors during the first 5 days (Table S3). (2) GAM analyses consistently demonstrated significantly lower platelet counts in non‐survivors than survivors over the initial 5 days across various subgroups. These subgroups included patients with more than three platelet measurements or those not receiving vasopressors at baseline (Supporting Figure 3); patients with NSTEMI or STEMI (Figure S4) patients aged < 65 years or ≥ 65 years (Figure S5); and a propensity score‐matched cohort (Figure S6). Central Illustration 1.

**Central Illustration 1:**
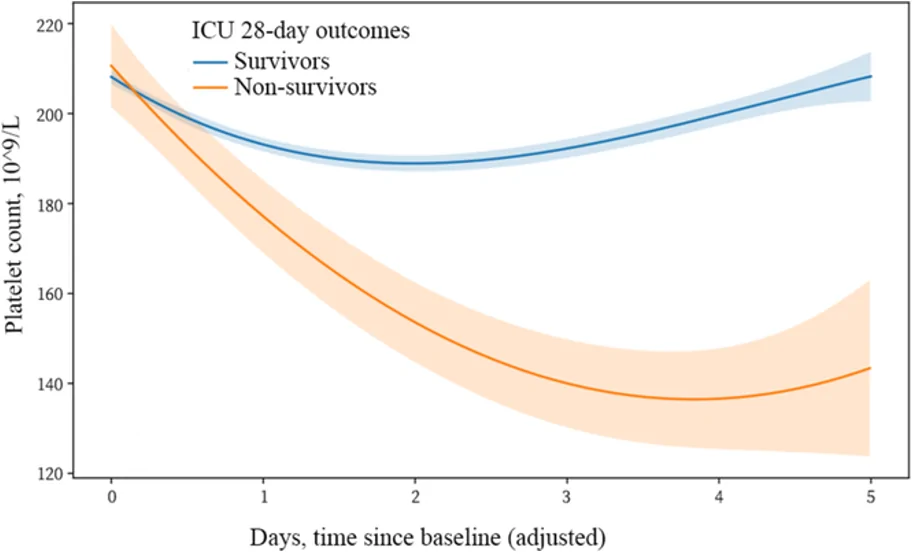
ICU 28‐day all‐cause mortality was 6.1% (410/6,769). During the first 5 days, platelet counts were significantly lower in ICU 28‐day non‐survivors than in survivors. The difference in platelet counts between ICU 28‐day non‐survivors and survivors increased by an average of 15.4×10^9^/L daily (95% CI, 14.1 to 16.7).

## 4 Discussion

This study investigated the dynamic changes in platelet count among AMI survivors and non‐survivors following ICU admission. The findings captured a general trend characterized by an initial decline and a subsequent increase in platelet count. Notably, during the first 5 days, the platelet counts were significantly lower in the non‐survivor group compared to the survivor group. Additionally, the difference in platelet count between the two groups widened over the first 5 days. The daily difference between non‐survivors and survivors increased by an average of 15.4 × 10^9^/L in the ICU 28‐day cohort, and by 18.0 × 10^9^/L in the ICU 7‐day cohort. An early and rapid decline in platelet count was associated with poor prognosis in patients with AMI.

The baseline mean platelet count was consistent with previous studies, which reported values ranging from 223.9 to 254.8 × 10^9^/L [23, 24, 25]. Furthermore, the platelet count in overall AMI patients exhibited a trend of an initial decrease and a subsequent increase, which was also consistent with previous studies [6, 17]. This study extended previous reports in several ways. First, it demonstrated the trend of platelet count over time after ICU admission stratified by ICU 7‐day and ICU 28‐day survival status. Second, the study found that the decrease in platelet count among non‐survivors was greater than that of survivors during the first 5 days. Third, the results showed that the early longitudinal changes in platelet count were associated with the clinical outcomes among AMI patients.

Several clinical factors may be associated with the observed longitudinal decrease in platelet count among patients with AMI. First, platelet activation and aggregation play critical roles in the thrombotic process of AMI, which may be accompanied by platelet count consumption and a longitudinal decrease in platelet count [26, 27, 28]. Second, reinfarction may be related to additional platelet count consumption and an associated longitudinal decline in platelet count. For instance, a study showed that in patients with STEMI, a greater decrease in follow‐up platelet count from baseline was independently associated with the risk of 30‐day reinfarction (odds ratio: 1.4, 95% CI: 1.1 to 1.8 for every 100 × 10^9^/L decrease) [25]. Third, infections may be associated with a longitudinal decrease in platelet count [8]. Fourth, illness severity and disease progression may be correlated with early longitudinal changes in platelet count [3, 29, 30, 31, 32]. However, due to the observational design of this study, it is impossible to infer a causal relationship between the early rapid longitudinal decline in platelet count and poor prognosis in AMI patients. The rapid decline in platelet count among non‑survivors may primarily reflect greater underlying disease severity, including larger infarct size, more profound systemic inflammation, and more extensive multiorgan dysfunction, rather than representing an independent causal pathway leading to death. The underlying pathophysiological mechanisms of the longitudinal decline in platelet count remain to be explored in future prospective studies.

Although the widening difference in platelet count between survivors and non‐survivors was statistically significant, its practical implications for risk stratification and clinical decision‐making remain unclear. Due to the observational design of this study, no prediction model was constructed or validated, and therefore the incremental prognostic value of longitudinal platelet measurements beyond established severity scores (e.g., SOFA) remains undetermined. While this study identified an independent association between early longitudinal platelet decline and ICU mortality, whether incorporating dynamic platelet count changes into existing risk models can significantly improve model performance remains to be validated. Serial platelet monitoring may aid in the early identification of high‐risk AMI patients in clinical practice, but its clinical utility warrants further exploration in prospective studies. Future studies may need to define clinically meaningful thresholds for the rate and magnitude of platelet count decline and evaluate whether incorporating longitudinal platelet changes into conventional prognostic tools can improve model performance.

This study had several limitations. First, the diagnosis of AMI relied on ICD‐9 codes without specific clinical adjudication (e.g., electrocardiogram or troponin trajectories), which might lead to misclassification, particularly in datasets like eICU‐CRD where direct validation was not feasible. Second, the inherent selection bias and unmeasured confounding in observational studies could not be fully eliminated. Although this study adjusted for numerous potential confounding factors, several unmeasured confounders were unavailable in the eICU‐CRD, and their confounding effects might still bias the observed associations: (1) infarct size: larger infarct size might be associated with greater platelet consumption [5], and might influence mortality [11]; (2) inflammatory status: higher levels of C‐reactive protein and procalcitonin might be associated with platelet activation and consumption [9, 33], and might exacerbate myocardial and multi‐organ injury; (3) bleeding severity: hemorrhagic complications might be associated with greater platelet loss, and might influence mortality [34]; (4) antithrombotic treatment intensity: higher anticoagulation dosing might be associated with drug‐related thrombocytopenia [35, 36], and might influence mortality; (5) arrhythmias: cardiac arrhythmias might be associated with platelet activation and a prothrombotic state through coagulation abnormalities [37], and might influence mortality [38]. Third, the classification of patients discharged from the ICU before the 7‐day or 28‐day endpoints as survivors left deaths occurring shortly after ICU discharge uncaptured, which might lead to underestimation of the true ICU 7‐day and ICU 28‐day mortality. Fourth, the inherent missingness of clinical data might affect the interpretation of the analysis; specifically, missing SOFA elements were assigned a score of 0, and unknown vasopressor doses [µg/(kg·min)] were categorized into the lowest tier, which might underestimate actual disease severity and introduce residual confounding. Fifth, because the study sample was predominantly Caucasian, the findings might not be applicable to other racial groups.

## 5 Conclusions

An early longitudinal decline in platelet count was associated with a poor prognosis in patients with AMI. However, this study is observational, and causality cannot be inferred.

## Authors Contributions

**Tianlai Lin and Jingqing Xu:** study design, data analysis, and manuscript preparation. **Xiaoling Zeng and Qiang Liu**: data analysis, literature research, and manuscript revision. **Mouying Liu and Xizhe Chen:** literature research and manuscript revision. **Youli Chen:** manuscript preparation and critical review.

## Consent

Not applicable.

## Ethics Statement

Ethics approval and informed consent for the eICU database were not applicable because it was released under the Health Insurance Portability and Accountability Act (HIPAA) safe harbor provision (HIPAA Certification No. 1031219‐2).

## Conflicts of Interest

The authors declare no conflicts of interest.

## Supporting information


Supporting File 1

Supporting File 2

Supporting File 3

Supporting File 4

Supporting File 5

Supporting File 6

Supporting File 7

Supporting File 8

Supporting File 9

Supporting File 10

Supporting File 11

Supporting File 12

Supporting File 13

Supporting File 14

Supporting File 15

Supporting File 16

## Data Availability

Data are fully available at https://eicu-crd.mit.edu.
