# Supplementary material for: An Early Longitudinal Decrease in Platelet Count Is Associated With Poor Prognosis in Acute Myocardial Infarction Patients: A Multicenter Retrospective Study
Source: Clin Cardiol. 2026 Oct 22;49(10):e70504. doi: 10.1002/clc.70504 (PMC13688426; doi:10.1002/clc.70504)
Supplement: Supplementary file 3 [file CLC-49-e70504-s008.pdf]

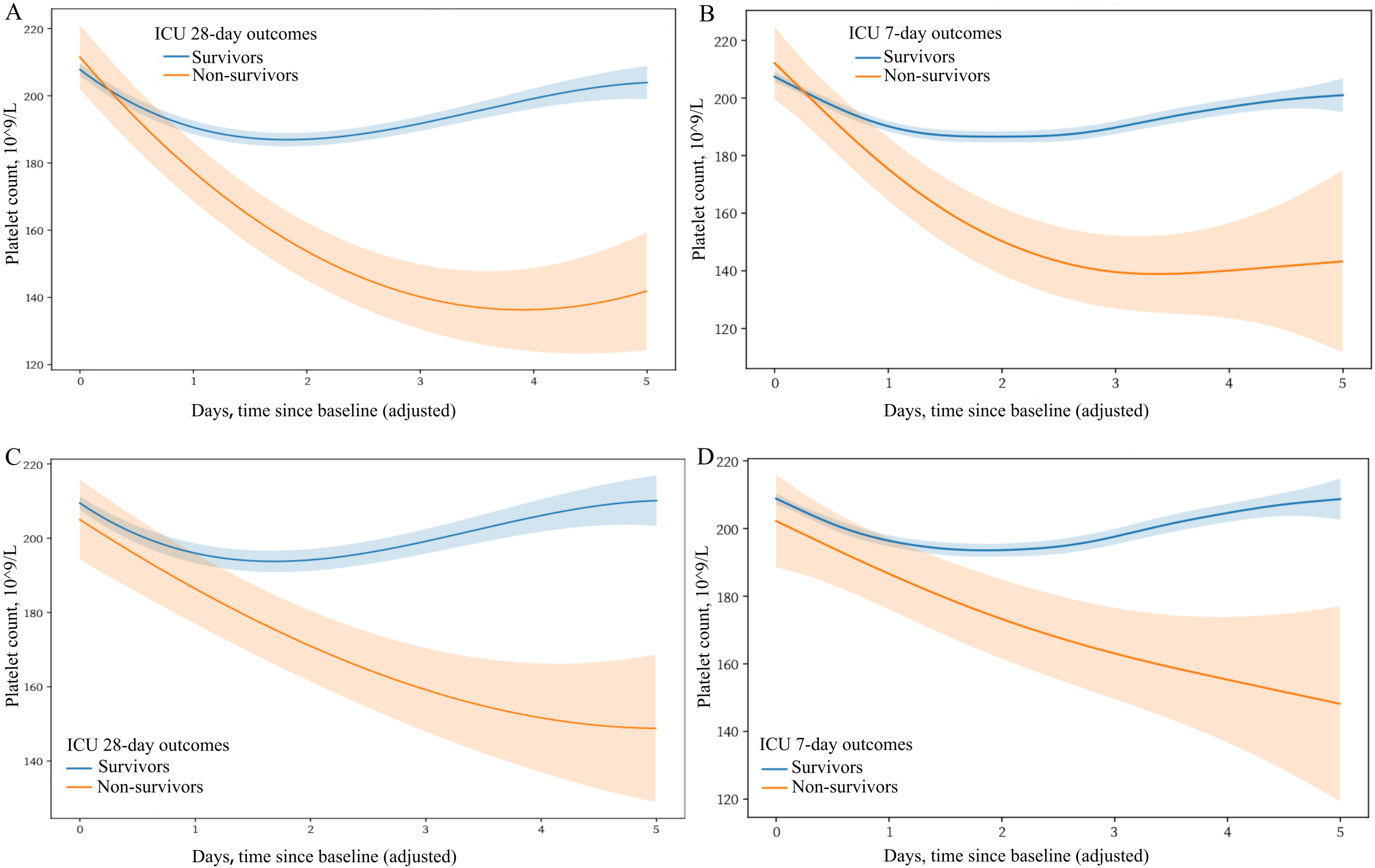

A
ICU 28-day outcomes
Survivors
Non-survivors
Platelet count, 10^9/L
220
200
180
160
140
120
0
1
2
3
4
5
Days, time since baseline (adjusted)
B
ICU 7-day outcomes
Survivors
Non-survivors
Platelet count, 10^9/L
220
200
180
160
140
120
0
1
2
3
4
5
Days, time since baseline (adjusted)
C
ICU 28-day outcomes
Survivors
Non-survivors
Platelet count, 10^9/L
220
200
180
160
140
0
1
2
3
4
5
Days, time since baseline (adjusted)
D
ICU 7-day outcomes
Survivors
Non-survivors
Platelet count, 10^9/L
220
200
180
160
140
120
0
1
2
3
4
5
Days, time since baseline (adjusted)
