# Supplementary material for: An Early Longitudinal Decrease in Platelet Count Is Associated With Poor Prognosis in Acute Myocardial Infarction Patients: A Multicenter Retrospective Study
Source: Clin Cardiol. 2026 Oct 22;49(10):e70504. doi: 10.1002/clc.70504 (PMC13688426; doi:10.1002/clc.70504)
Supplement: Supplementary file 5 [file CLC-49-e70504-s012.pdf]

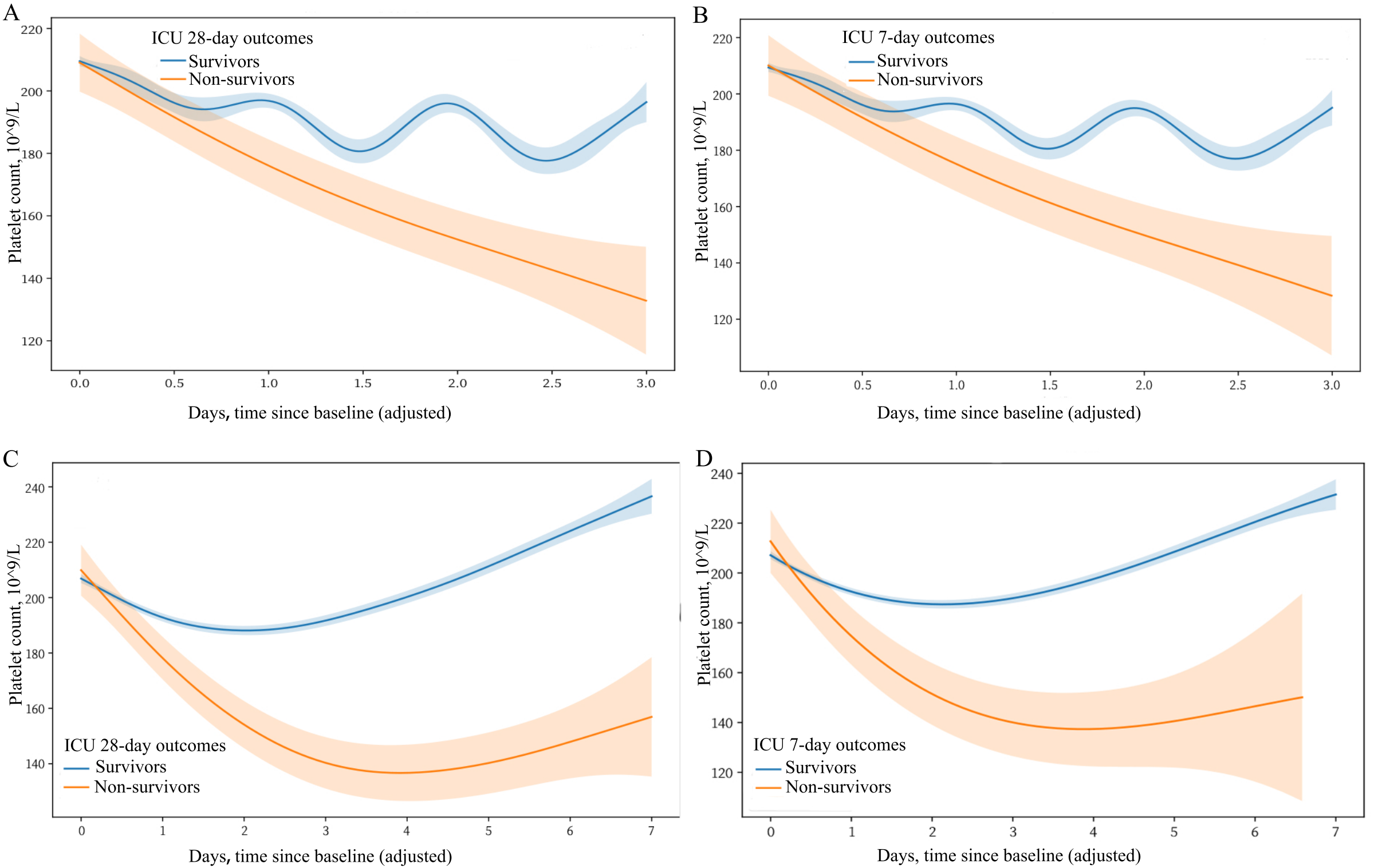

A
ICU 28-day outcomes
Survivors
Non-survivors
Platelet count, 10^9/L
220
200
180
160
140
120
0.0
0.5
1.0
1.5
2.0
2.5
3.0
Days, time since baseline (adjusted)
B
ICU 7-day outcomes
Survivors
Non-survivors
Platelet count, 10^9/L
220
200
180
160
140
120
0.0
0.5
1.0
1.5
2.0
2.5
3.0
Days, time since baseline (adjusted)
C
ICU 28-day outcomes
Survivors
Non-survivors
Platelet count, 10^9/L
240
220
200
180
160
140
0
1
2
3
4
5
6
7
Days, time since baseline (adjusted)
D
ICU 7-day outcomes
Survivors
Non-survivors
Platelet count, 10^9/L
240
220
200
180
160
140
120
0
1
2
3
4
5
6
7
Days, time since baseline (adjusted)
