# Supplementary material for: An Early Longitudinal Decrease in Platelet Count Is Associated With Poor Prognosis in Acute Myocardial Infarction Patients: A Multicenter Retrospective Study
Source: Clin Cardiol. 2026 Oct 22;49(10):e70504. doi: 10.1002/clc.70504 (PMC13688426; doi:10.1002/clc.70504)
Supplement: Supplementary file 7 [file CLC-49-e70504-s001.docx]

**Supplementary Table 3. Association between platelet counts within the first 5 days and prognosis across different prognostic timeframes**

| **Prognostic Timeframe** | **Group** | **β (95% CI)** | **P-value** |
| --- | --- | --- | --- |
| Primary:ICU 28-day mortality | Survivors | -1.9 (-2.7, -1.1) | <0.001 |
|  | Non-survivors | -16.6 (-20.1, -13.2) | <0.001 |
| Secondary: ICU 7-day mortality | Survivors | -2.4 (-3.3, -1.6) | <0.001 |
|  | Non-survivors | -19.1 (-24.5, -13.8) | <0.001 |

Adjusted for adjusted for age, gender, ethnicity, body mass index, temperature, mean arterial pressure, Glasgow Coma Scale score, SOFA, Acute Physiology Score III, diabetes mellitus, chronic heart failure, pneumonia, sepsis, stroke, bleeding, hemorrhage, blood urea nitrogen, serum creatinine, red blood cell count, white blood cell count, lactate, microbiological culture, dobutamine, dopamine, epinephrine, norepinephrine, aspirin, clopidogrel, heparin, enoxaparin, warfarin, thrombolytic therapy, mechanical ventilation, PTCA.
