# Supplementary material for: An Early Longitudinal Decrease in Platelet Count Is Associated With Poor Prognosis in Acute Myocardial Infarction Patients: A Multicenter Retrospective Study
Source: Clin Cardiol. 2026 Oct 22;49(10):e70504. doi: 10.1002/clc.70504 (PMC13688426; doi:10.1002/clc.70504)
Supplement: Supplementary file 9 [file CLC-49-e70504-s004.docx]

**Supplementary Table1. Extraction of variables from the eICU-CRD**

| **Category** | **Variables** | **Table** |
| --- | --- | --- |
| Demographics and ICU outcomes | age, gender, ethnicity, weight, height, ICU discharge offset, ICU discharge status | Patient |
| Vital signs and score | temperature, mean arterial pressure, Glasgow Coma Scale score | Apacheapsvar |
| Comorbidities and complications | diabetes mellitus, chronic heart failure, pneumonia, stroke, bleeding, hemorrhage, HIT | Diagnosis |
| Initial laboratory values (first ICU 24 hours) | blood urea nitrogen, serum creatinine, red blood cell count, hemoglobin, white blood cell count, lactate | Lab |
| Microbiological culture results | microbiological culture | Microlab |
| Vasoactive agents (first ICU 24 hours) | dobutamine, dopamine, epinephrine, norepinephrine | Infusiondrug |
| Medications | aspirin, clopidogrel, heparin, enoxaparin, warfarin | Medication |
| Treatments | platelet concentrate transfusion, thrombolytic therapy | Treatment |
| Interventions and score | Acute Physiology Score, mechanical ventilation, PTCA | Apachepatientresult |

ICU, intensive care unit; eICU-CRD, eICU Collaborative Research Database; HIT, heparin-induced thrombocytopenia; PTCA, percutaneous transluminal coronary angioplasty.
