# Supplementary material for: An Early Longitudinal Decrease in Platelet Count Is Associated With Poor Prognosis in Acute Myocardial Infarction Patients: A Multicenter Retrospective Study
Source: Clin Cardiol. 2026 Oct 22;49(10):e70504. doi: 10.1002/clc.70504 (PMC13688426; doi:10.1002/clc.70504)
Supplement: Supplementary file 11 [file CLC-49-e70504-s006.docx]

**Supplementary Figures 6. Changes in platelet count over time in AMI non-survivors and survivors after propensity score matching.**

**A: ICU 28-day outcome: adjusted model; B: ICU 7-day outcome: adjusted model.**

Adjustment variables include: age, gender, ethnicity, body mass index, temperature, mean arterial pressure, Glasgow Coma Scale score, SOFA, Acute Physiology Score III, diabetes mellitus, chronic heart failure, pneumonia, sepsis, stroke, bleeding, hemorrhage, blood urea nitrogen, serum creatinine, red blood cell count, white blood cell count, lactate, microbiological culture, dobutamine, dopamine, epinephrine, norepinephrine, aspirin, clopidogrel, heparin, enoxaparin, warfarin, thrombolytic therapy, mechanical ventilation, and PTCA.
