# Supplementary material for: An Early Longitudinal Decrease in Platelet Count Is Associated With Poor Prognosis in Acute Myocardial Infarction Patients: A Multicenter Retrospective Study
Source: Clin Cardiol. 2026 Oct 22;49(10):e70504. doi: 10.1002/clc.70504 (PMC13688426; doi:10.1002/clc.70504)
Supplement: Supplementary file 12 [file CLC-49-e70504-s009.docx]

**Supplementary Table 2. Multicollinearity screening of independent variables**

| **Variable** | **VIF** |
| --- | --- |
| Age | 1.3 |
| Gender | 1.1 |
| Ethnicity | 1.1 |
| Body mass index | 1.1 |
| Temperature | 1.5 |
| Mean arterial pressure | 1.1 |
| Glasgow Coma Scale score | 2.1 |
| SOFA | 4.6 |
| Acute Physiology Score III | 4.2 |
| Diabetes mellitus | 1.2 |
| Chronic heart failure | 1.1 |
| Pneumonia | 1.2 |
| Sepsis | 1.3 |
| Stroke | 1.1 |
| Bleeding | 1.2 |
| Hemorrhage | 1.1 |
| Blood urea nitrogen | 2.2 |
| Serum creatinine | 2.3 |
| Serum potassium | 1.2 |
| Red blood cell count | 5.1 |
| Hemoglobin | 5.2 |
| White blood cell count | 1.1 |
| Lactate | 1.4 |
| Microbiological culture | 1.1 |
| Dobutamine | 1.1 |
| Dopamine | 1.1 |
| Epinephrine | 1.2 |
| Norepinephrine | 1.7 |
| Aspirin | 1.6 |
| Clopidogrel | 1.2 |
| Heparin | 1.4 |
| Enoxaparin | 1.2 |
| Warfarin | 1.1 |
| Thrombolytic therapy | 1 |
| Mechanical ventilation | 1.6 |
| PTCA | 1.3 |

VIF, variance inflation factor; SOFA, Sequential Organ Failure Assessment; PTCA, percutaneous transluminal coronary angioplasty.
