# Supplementary material for: An Early Longitudinal Decrease in Platelet Count Is Associated With Poor Prognosis in Acute Myocardial Infarction Patients: A Multicenter Retrospective Study
Source: Clin Cardiol. 2026 Oct 22;49(10):e70504. doi: 10.1002/clc.70504 (PMC13688426; doi:10.1002/clc.70504)
Supplement: Supplementary file 14 [file CLC-49-e70504-s014.docx]

**Supplementary Material:**

**R script: Fully adjusted GAMM for early platelet changes (within 5 days) in patients with AMI stratified by ICU 28-day mortality**

\## Created by EmpowerStats @ Sun, 15 Mar 26 14:41:14 +0100##

#******************** Regarding ALL Following R Code	********************

#*** COPYRIGHT (c) 2010, 2021 X&Y Solutions, ALL RIGHT RESERVED ***********

#******************* www.EmpowerStats.com *********************************

#**************************************************************************

Sys.setlocale(category = 'LC_ALL', locale = 'English_United States.1252');

.libPaths(file.path(R.home(),'library'));

library(doBy);

options(timeout=600);

library(plotrix);

library(stringi);

library(stringr);

library(survival);

library(rms);

library(nnet);

library(car);

library(mgcv);

pdfwd<-6; pdfht<-6;

load('C:/EmpowerXYS/A123/Analysis/eICU/AMIfx2/PLATELETS.Rdata');

if (length(which(ls()=='EmpowerStatsR'))==0) EmpowerStatsR<-get(ls()[1]);

names(EmpowerStatsR)<-toupper(names(EmpowerStatsR));

originalVNAME<-names(EmpowerStatsR);

ofname<-'AMIfx2_59_tbl';

recodevar <- function (var,oldcode,newcode) {

tmp.v <- var

nc.tmp <- length(oldcode)

str <- 0;

if (!is.numeric(oldcode)) {

if (sum(grepl('[|(|)|]|^|*|+|$|.|\\|/', oldcode))==0) {

if (sum(duplicated(substr(oldcode,1,min(nchar(oldcode)))))==0) {oldcode<-paste("^",oldcode,sep=""); str <- 1;}

}

}

for (i in (1:nc.tmp)) {

if (!is.na(oldcode[i]) & oldcode[i] != "NA") {

if (str == 1) {tmp.v[str_detect(var, str_escape('(0,19]'))] <- newcode[i];

} else {tmp.v[var == oldcode[i]]<-newcode[i]; }

} else if (!is.na(newcode[i])) {tmp.v[is.na(var)] <- newcode[i] }

}

if (is.factor(tmp.v)) {tmp.v1<-as.numeric(as.character(tmp.v))} else {tmp.v1<-as.numeric(tmp.v)}

rm(tmp.v); return(tmp.v1)

}

attach(EmpowerStatsR);

sink(paste(ofname,'_datastep.lst',sep=''));

print('Creating new variable: NEWTIME');

NEWTIME<-TIME/1440;

summary(NEWTIME);

EmpowerStatsR<-cbind(EmpowerStatsR,NEWTIME);

print('Creating new variable: BMINEW');

BMINEW<-rep(NA, times=nrow(EmpowerStatsR));

tmp <- is.na(BMINEW) & ((!is.na(BMI) & (BMI > 0)));

tmp <- (!is.na(tmp) & tmp);

BMINEW[tmp]<-(BMI)[tmp];

BMINEW[is.na(BMINEW)]<-0;

summary(BMINEW);

EmpowerStatsR<-cbind(EmpowerStatsR,BMINEW);

print('Creating new variable: BMIG');

BMIG<-rep(NA, times=nrow(EmpowerStatsR));

tmp <- is.na(BMIG) & ((!is.na(BMI) & (BMI > 0)));

tmp <- (!is.na(tmp) & tmp);

BMIG[tmp]<-0;

BMIG[is.na(BMIG)]<-1;

summary(BMIG);

EmpowerStatsR<-cbind(EmpowerStatsR,BMIG);

print('Creating new variable: LACNEW');

LACNEW<-rep(NA, times=nrow(EmpowerStatsR));

tmp <- is.na(LACNEW) & ((!is.na(LAC) & (LAC > 0)));

tmp <- (!is.na(tmp) & tmp);

LACNEW[tmp]<-(BMI)[tmp];

LACNEW[is.na(LACNEW)]<-0;

summary(LACNEW);

EmpowerStatsR<-cbind(EmpowerStatsR,LACNEW);

print('Creating new variable: LACG');

LACG<-rep(NA, times=nrow(EmpowerStatsR));

tmp <- is.na(LACG) & ((!is.na(LAC) & (LAC > 0)));

tmp <- (!is.na(tmp) & tmp);

LACG[tmp]<-0;

LACG[is.na(LACG)]<-1;

summary(LACG);

EmpowerStatsR<-cbind(EmpowerStatsR,LACG);

print('Creating new variable: APNEW');

APNEW<-rep(NA, times=nrow(EmpowerStatsR));

tmp <- is.na(APNEW) & ((!is.na(ACUTEPHYSIOLOGYSCORE) & (ACUTEPHYSIOLOGYSCORE > 0)));

tmp <- (!is.na(tmp) & tmp);

APNEW[tmp]<-(ACUTEPHYSIOLOGYSCORE)[tmp];

APNEW[is.na(APNEW)]<-0;

summary(APNEW);

EmpowerStatsR<-cbind(EmpowerStatsR,APNEW);

print('Creating new variable: APG');

APG<-rep(NA, times=nrow(EmpowerStatsR));

tmp <- is.na(APG) & ((!is.na(ACUTEPHYSIOLOGYSCORE) & (ACUTEPHYSIOLOGYSCORE > 0)));

tmp <- (!is.na(tmp) & tmp);

APG[tmp]<-0;

APG[is.na(APG)]<-1;

summary(APG);

EmpowerStatsR<-cbind(EmpowerStatsR,APG);

print('Creating new variable: TNEW');

TNEW<-rep(NA, times=nrow(EmpowerStatsR));

tmp <- is.na(TNEW) & ((!is.na(TEMPERATURE) & (TEMPERATURE > 0)));

tmp <- (!is.na(tmp) & tmp);

TNEW[tmp]<-(TEMPERATURE)[tmp];

TNEW[is.na(TNEW)]<-0;

summary(TNEW);

EmpowerStatsR<-cbind(EmpowerStatsR,TNEW);

print('Creating new variable: TG');

TG<-rep(NA, times=nrow(EmpowerStatsR));

tmp <- is.na(TG) & ((!is.na(TEMPERATURE) & (TEMPERATURE > 0)));

tmp <- (!is.na(tmp) & tmp);

TG[tmp]<-0;

TG[is.na(TG)]<-1;

summary(TG);

EmpowerStatsR<-cbind(EmpowerStatsR,TG);

print('Creating new variable: BPNEW');

BPNEW<-rep(NA, times=nrow(EmpowerStatsR));

tmp <- is.na(BPNEW) & ((!is.na(MEANBP) & (MEANBP > 0)));

tmp <- (!is.na(tmp) & tmp);

BPNEW[tmp]<-(MEANBP)[tmp];

BPNEW[is.na(BPNEW)]<-0;

summary(BPNEW);

EmpowerStatsR<-cbind(EmpowerStatsR,BPNEW);

print('Creating new variable: BPG');

BPG<-rep(NA, times=nrow(EmpowerStatsR));

tmp <- is.na(BPG) & ((!is.na(MEANBP) & (MEANBP > 0)));

tmp <- (!is.na(tmp) & tmp);

BPG[tmp]<-0;

BPG[is.na(BPG)]<-1;

summary(BPG);

EmpowerStatsR<-cbind(EmpowerStatsR,BPG);

print('Creating new variable: BUNNEW');

BUNNEW<-rep(NA, times=nrow(EmpowerStatsR));

tmp <- is.na(BUNNEW) & ((!is.na(BUN) & (BUN > 0)));

tmp <- (!is.na(tmp) & tmp);

BUNNEW[tmp]<-(BUN)[tmp];

BUNNEW[is.na(BUNNEW)]<-0;

summary(BUNNEW);

EmpowerStatsR<-cbind(EmpowerStatsR,BUNNEW);

print('Creating new variable: BUNG');

BUNG<-rep(NA, times=nrow(EmpowerStatsR));

tmp <- is.na(BUNG) & ((!is.na(BUN) & (BUN > 0)));

tmp <- (!is.na(tmp) & tmp);

BUNG[tmp]<-0;

BUNG[is.na(BUNG)]<-1;

summary(BUNG);

EmpowerStatsR<-cbind(EmpowerStatsR,BUNG);

print('Creating new variable: CRNEW');

CRNEW<-rep(NA, times=nrow(EmpowerStatsR));

tmp <- is.na(CRNEW) & ((!is.na(CR) & (CR > 0)));

tmp <- (!is.na(tmp) & tmp);

CRNEW[tmp]<-(CR)[tmp];

CRNEW[is.na(CRNEW)]<-0;

summary(CRNEW);

EmpowerStatsR<-cbind(EmpowerStatsR,CRNEW);

print('Creating new variable: CRG');

CRG<-rep(NA, times=nrow(EmpowerStatsR));

tmp <- is.na(CRG) & ((!is.na(CR) & (CR > 0)));

tmp <- (!is.na(tmp) & tmp);

CRG[tmp]<-0;

CRG[is.na(CRG)]<-1;

summary(CRG);

EmpowerStatsR<-cbind(EmpowerStatsR,CRG);

print('Creating new variable: RBCNEW');

RBCNEW<-rep(NA, times=nrow(EmpowerStatsR));

tmp <- is.na(RBCNEW) & ((!is.na(RBC) & (RBC > 0)));

tmp <- (!is.na(tmp) & tmp);

RBCNEW[tmp]<-(RBC)[tmp];

RBCNEW[is.na(RBCNEW)]<-0;

summary(RBCNEW);

EmpowerStatsR<-cbind(EmpowerStatsR,RBCNEW);

print('Creating new variable: WBCNEW');

WBCNEW<-rep(NA, times=nrow(EmpowerStatsR));

tmp <- is.na(WBCNEW) & ((!is.na(WBC) & (WBC > 0)));

tmp <- (!is.na(tmp) & tmp);

WBCNEW[tmp]<-(WBC)[tmp];

WBCNEW[is.na(WBCNEW)]<-0;

summary(WBCNEW);

EmpowerStatsR<-cbind(EmpowerStatsR,WBCNEW);

print('Creating new variable: WBCG');

WBCG<-rep(NA, times=nrow(EmpowerStatsR));

tmp <- is.na(WBCG) & ((!is.na(WBC) & (WBC > 0)));

tmp <- (!is.na(tmp) & tmp);

WBCG[tmp]<-0;

WBCG[is.na(WBCG)]<-1;

summary(WBCG);

EmpowerStatsR<-cbind(EmpowerStatsR,WBCG);

print('Creating new variable: GCS.CONT');

GCS.CONT<- GCS;

summary(GCS.CONT);

EmpowerStatsR<-cbind(EmpowerStatsR,GCS.CONT);

print('Creating new variable: GCS.CONT.C');

GCS.CONT.C<- 0+(GCS.CONT>8);

GCS.CONT.C[is.na(GCS.CONT)]<- NA;

summary(GCS.CONT.C);

EmpowerStatsR<-cbind(EmpowerStatsR,GCS.CONT.C);

print('Creating new variable: GCS.CONT.C.RCD');

GCS.CONT.C.RCD<- recodevar(GCS.CONT.C,c(0,1,NA),c(0,1,2));

summary(GCS.CONT.C.RCD);

EmpowerStatsR<-cbind(EmpowerStatsR,GCS.CONT.C.RCD);

print('Creating new variable: ETHNICITY.RCD');

ETHNICITY.RCD<- recodevar(ETHNICITY,c(0,1,2,3,4,5),c(0,1,2,2,2,2));

summary(ETHNICITY.RCD);

EmpowerStatsR<-cbind(EmpowerStatsR,ETHNICITY.RCD);

print('Creating new variable: SOFA_TOTAL.CONT');

SOFA_TOTAL.CONT<- SOFA_TOTAL;

summary(SOFA_TOTAL.CONT);

EmpowerStatsR<-cbind(EmpowerStatsR,SOFA_TOTAL.CONT);

rm(NEWTIME,BMINEW,BMIG,LACNEW,LACG,APNEW,APG,TNEW,TG,BPNEW,BPG,BUNNEW,BUNG,CRNEW,CRG,RBCNEW,WBCNEW,WBCG,GCS.CONT,GCS.CONT.C,GCS.CONT.C.RCD,ETHNICITY.RCD,SOFA_TOTAL.CONT);

sink();

vname<-c(NA,'PATIENTUNITSTAYID','TIME','PLATELETS','GENDER','GENDER.0','GENDER.1','AGE','ETHNICITY','ETHNICITY.0','ETHNICITY.1','ETHNICITY.2','ETHNICITY.3','ETHNICITY.4','ETHNICITY.5','BMI','DEATH28_UNIT','DEATH28_UNIT.0','DEATH28_UNIT.1','GCS','GCS.3','GCS.4','GCS.5','GCS.6','GCS.7','GCS.8','GCS.9','GCS.10','GCS.11','GCS.12','GCS.13','GCS.14','GCS.15','TEMPERATURE','MEANBP','SOFA_TOTAL','SOFA_TOTAL.0','SOFA_TOTAL.1','SOFA_TOTAL.2','SOFA_TOTAL.3','SOFA_TOTAL.4','SOFA_TOTAL.5','SOFA_TOTAL.6','SOFA_TOTAL.7','SOFA_TOTAL.8','SOFA_TOTAL.9','SOFA_TOTAL.10','SOFA_TOTAL.11','SOFA_TOTAL.12','SOFA_TOTAL.13','SOFA_TOTAL.14','SOFA_TOTAL.15','SOFA_TOTAL.16','SOFA_TOTAL.17','ACUTEPHYSIOLOGYSCORE','CHF','CHF.0','CHF.1','DM','DM.0','DM.1','PNEUMONIA','PNEUMONIA.0','PNEUMONIA.1','BLEEDING','BLEEDING.0','BLEEDING.1','HEMORRHAGE','HEMORRHAGE.0','HEMORRHAGE.1','BUN','CR','RBC','WBC','LAC','PEIYANG','PEIYANG.0','PEIYANG.1','STROKE','STROKE.0','STROKE.1','SOFA_DOBUTAMINE','SOFA_DOBUTAMINE.0','SOFA_DOBUTAMINE.2','SOFA_DOPAMINE','SOFA_DOPAMINE.0','SOFA_DOPAMINE.2','SOFA_DOPAMINE.3','SOFA_DOPAMINE.4','SOFA_DOPAMINE.9','SOFA_EPINEPHRINE','SOFA_EPINEPHRINE.0','SOFA_EPINEPHRINE.3','SOFA_EPINEPHRINE.4','SOFA_EPINEPHRINE.9','SOFA_NOREPINEPHRINE','SOFA_NOREPINEPHRINE.0','SOFA_NOREPINEPHRINE.3','SOFA_NOREPINEPHRINE.4','SOFA_NOREPINEPHRINE.9','ASPIRIN','ASPIRIN.0','ASPIRIN.1','CLOPIDOGREL','CLOPIDOGREL.0','CLOPIDOGREL.1','ENOXAPARIN','ENOXAPARIN.0','ENOXAPARIN.1','HEPARIN','HEPARIN.0','HEPARIN.1','WARFARIN','WARFARIN.0','WARFARIN.1','PTCAWITHIN24H','PTCAWITHIN24H.0','PTCAWITHIN24H.1','MV','MV.0','MV.1','INFARCTIONTHROMBOLYTIC_THERAPY','INFARCTIONTHROMBOLYTIC_THERAPY.0','INFARCTIONTHROMBOLYTIC_THERAPY.1','SEPSIS_DIAGNOSIS','SEPSIS_DIAGNOSIS.0','SEPSIS_DIAGNOSIS.1','NEWTIME','BMINEW','BMIG','BMIG.0','BMIG.1','LACNEW','LACG','LACG.0','LACG.1','APNEW','APG','APG.0','APG.1','TNEW','TG','TG.0','TG.1','BPNEW','BPG','BPG.0','BPG.1','BUNNEW','BUNG','BUNG.0','BUNG.1','CRNEW','CRG','CRG.0','CRG.1','RBCNEW','WBCNEW','WBCG','WBCG.0','WBCG.1','GCS.CONT','GCS.CONT.C','GCS.CONT.C.0','GCS.CONT.C.1','GCS.CONT.C.RCD','GCS.CONT.C.RCD.0','GCS.CONT.C.RCD.1','GCS.CONT.C.RCD.2','ETHNICITY.RCD','ETHNICITY.RCD.0','ETHNICITY.RCD.1','ETHNICITY.RCD.2','SOFA_TOTAL.CONT')[-1];

vlabel<-c(NA,'PATIENTUNITSTAYID','TIME','PLATELETS','GENDER',' 0',' 1','AGE','ETHNICITY',' 0',' 1',' 2',' 3',' 4',' 5','BMI','DEATH28_UNIT',' 0',' 1','GCS',' 3',' 4',' 5',' 6',' 7',' 8',' 9',' 10',' 11',' 12',' 13',' 14',' 15','TEMPERATURE','MEANBP','SOFA_TOTAL',' 0',' 1',' 2',' 3',' 4',' 5',' 6',' 7',' 8',' 9',' 10',' 11',' 12',' 13',' 14',' 15',' 16',' 17','ACUTEPHYSIOLOGYSCORE','CHF',' 0',' 1','DM',' 0',' 1','PNEUMONIA',' 0',' 1','BLEEDING',' 0',' 1','HEMORRHAGE',' 0',' 1','BUN','CR','RBC','WBC','LAC','PEIYANG',' 0',' 1','STROKE',' 0',' 1','SOFA_DOBUTAMINE',' 0',' 2','SOFA_DOPAMINE',' 0',' 2',' 3',' 4',' 9','SOFA_EPINEPHRINE',' 0',' 3',' 4',' 9','SOFA_NOREPINEPHRINE',' 0',' 3',' 4',' 9','ASPIRIN',' 0',' 1','CLOPIDOGREL',' 0',' 1','ENOXAPARIN',' 0',' 1','HEPARIN',' 0',' 1','WARFARIN',' 0',' 1','PTCAWITHIN24H',' 0',' 1','MV',' 0',' 1','INFARCTIONTHROMBOLYTIC_THERAPY',' 0',' 1','SEPSIS_DIAGNOSIS',' 0',' 1','NEWTIME','BMINEW','BMIG',' 0',' 1','LACNEW','LACG',' 0',' 1','APNEW','APG',' 0',' 1','TNEW','TG',' 0',' 1','BPNEW','BPG',' 0',' 1','BUNNEW','BUNG',' 0',' 1','CRNEW','CRG',' 0',' 1','RBCNEW','WBCNEW','WBCG',' 0',' 1','GCS continuous','GCS continuous categorical',' <=8',' >8','GCS continuous categorical recoded',' 0',' 1',' 2','ETHNICITY recoded',' 0',' 1',' 2','SOFA_TOTAL continuous')[-1];

varused4this <- c('PATIENTUNITSTAYID','TIME','PLATELETS','GENDER','AGE','ETHNICITY','BMI','DEATH28_UNIT','GCS','TEMPERATURE','MEANBP','SOFA_TOTAL','ACUTEPHYSIOLOGYSCORE','CHF','DM','PNEUMONIA','BLEEDING','HEMORRHAGE','BUN','CR','RBC','WBC','LAC','PEIYANG','STROKE','SOFA_DOBUTAMINE','SOFA_DOPAMINE','SOFA_EPINEPHRINE','SOFA_NOREPINEPHRINE','ASPIRIN','CLOPIDOGREL','ENOXAPARIN','HEPARIN','WARFARIN','PTCAWITHIN24H','MV','INFARCTIONTHROMBOLYTIC_THERAPY','SEPSIS_DIAGNOSIS','NEWTIME','BMINEW','BMIG','LACNEW','LACG','APNEW','APG','TNEW','TG','BPNEW','BPG','BUNNEW','BUNG','CRNEW','CRG','RBCNEW','WBCNEW','WBCG','GCS.CONT','GCS.CONT.C','GCS.CONT.C.RCD','ETHNICITY.RCD','SOFA_TOTAL.CONT');

pkgs<-c('mgcv','nlme','lme4','gdata','nortest');

for (g in pkgs) {

if (!(g %in% rownames(installed.packages()))) install.packages(g,repos='https://cloud.r-project.org');

}

library(mgcv);

library(nlme);

library(lme4);

library(gdata);

library(nortest);

WD <- EmpowerStatsR; rm(EmpowerStatsR); gc();

title<-'广义相加混合模型';

WD<-subset(WD, ((!is.na(NEWTIME) & (NEWTIME < 5))));

wd.subset<-paste("Use subset of data: (!is.na(NEWTIME) & (NEWTIME < 5))");

weights.var<- NA;

yvname<-c('PLATELETS');

ydist<-c('gaussian');

ylink<-c('identity');

ylv<-c(0);

vname.start<-'PATIENTUNITSTAYID'; tstartlv<- 0;

xvname<-c('NEWTIME','AGE','GENDER','ETHNICITY.RCD','BMINEW','BMIG','GCS.CONT.C.RCD','TNEW','TG','BPNEW','BPG','SOFA_TOTAL.CONT','APNEW','APG','DM','CHF','PNEUMONIA','SEPSIS_DIAGNOSIS','STROKE','BLEEDING','HEMORRHAGE','PEIYANG','SOFA_DOBUTAMINE','SOFA_DOPAMINE','SOFA_EPINEPHRINE','SOFA_NOREPINEPHRINE','ASPIRIN','CLOPIDOGREL','ENOXAPARIN','HEPARIN','WARFARIN','INFARCTIONTHROMBOLYTIC_THERAPY','MV','PTCAWITHIN24H','BUNNEW','BUNG','CRNEW','CRG','WBCNEW','WBCG','LACNEW','LACG','RBCNEW');

sxf<-c('S','0','0','0','0','0','0','0','0','0','0','0','0','0','0','0','0','0','0','0','0','0','0','0','0','0','0','0','0','0','0','0','0','0','0','0','0','0','0','0','0','0','0');

xlv<-c(0,0,2,3,0,2,3,0,2,0,2,0,0,2,2,2,2,2,2,2,2,2,2,5,4,4,2,2,2,2,2,2,2,2,0,2,0,2,0,2,0,2,0);

svname<-c();

vname.stop<- NA;

chk<- 1;

colvname<- NA;

bvar<-'DEATH28_UNIT'; blv<- 2;

dec<-4;

##R package## mgcv nlme lme4 gdata nortest ##R package##;

pvformat<-function(p,dec) {

pp <- sprintf(paste("%.",dec,"f",sep=""),as.numeric(p))

if (is.matrix(p)) {pp<-matrix(pp, nrow=nrow(p)); colnames(pp)<-colnames(p);rownames(pp)<-rownames(p);}

lw <- paste("<",substr("0.00000000000",1,dec+1),"1",sep="");

pp[as.numeric(p)<(1/10^dec)]<-lw

return(pp)

}

numfmt<-function(p,dec) {

if (is.list(p)) p<-as.matrix(p)

pp <- sprintf(paste("%.",dec,"f",sep=""),as.numeric(p))

if (is.matrix(p)) {pp<-matrix(pp, nrow=nrow(p));colnames(pp)<-colnames(p);rownames(pp)<-rownames(p);}

pp[as.numeric(p)>10000000]<- "inf."

pp[is.na(p) | gsub(" ","",p)==""]<- ""

pp[p=="-Inf"]<-"-Inf"

pp[p=="Inf"]<-"Inf"

return(pp)

}

printTxt<-function(txt,cname,rname) {

tmp<-as.matrix(txt,ncol=1); rname1<-rname

if (length(rname1)==1) rname1=rep(rname,times=nrow(tmp))

colnames(tmp)=cname; rownames(tmp)=rname1; print(tmp,quote=F)

}

getNumber<-function(str, n) {

str<-substr(str,2,nchar(str)-1)

for (i in (1:nchar(str))) {if (substr(str,i,i)==",") {p=i; break}; }

ifelse(n==1,return(substr(str,1,p-1)),return(substr(str,p+1,nchar(str))))

}

legLocate<-function(x,y) {

xmin<-min(x,na.rm=TRUE); xmax<-max(x,na.rm=TRUE)

ymin<-min(y,na.rm=TRUE); ymax<-max(y,na.rm=TRUE)

yoff<-(ymax-ymin); tmp<-table(cut(x,3),cut(y,4))

tmp.r=which.min(tmp[,4]);tmp.c=4

if (tmp[2,1]==0) {tmp.r=2;tmp.c=1}

if (tmp[1,1]==0) {tmp.r=1;tmp.c=1}

if (tmp[3,1]==0) {tmp.r=3;tmp.c=1}

if (tmp[2,4]==0) {tmp.r=2;tmp.c=4}

if (tmp[1,4]==0) {tmp.r=1;tmp.c=4}

if (tmp[3,4]==0) {tmp.r=3;tmp.c=4}

pos.y<-colnames(tmp)[tmp.c]; pos.x<-rownames(tmp)[tmp.r]; pct<-0.15

if (tmp.c==4) {

if (min(tmp[,4])>0) {pct<-0.3}

ymax<-ymax+yoff*pct; legy<-ymax;ymin<-ymin-yoff*0.1

}

if (tmp.c==1) {

if (min(tmp[,1])>0) {pct<-0.3}

legy<-as.numeric(getNumber(pos.y,2));ymin<-ymin-yoff*pct;ymax=ymax+yoff*0.1

}

legx<-as.numeric(getNumber(pos.x,1))

return(cbind(xmin,xmax,ymin,ymax,legx,legy))

}

mat2htmltable<-function(mat) {

t1<- apply(mat,1,function(z) paste(z,collapse="</td><td>"))

t2<- paste("<tr><td>",t1,"</td></tr>")

return(paste(t2,collapse=" "))

}

lme2htmltable<-function(mdl) {

gm<-summary(mdl); print(gm)

if (is.list(gm)) {

coe<-gm$tTable

if (!is.null(coe)) {

cnames<-c(colnames(coe),"95%CI.low","95%CI.upp")

coe<-cbind(coe,coe[,1]-1.96*coe[,2],coe[,1]+1.96*coe[,2])

coe[,c(1,2,4,6,7)]<-numfmt(coe[,c(1,2,4,6,7)],dec)

coe[,5]<-pvformat(coe[,5],dec)

oo1<-cbind(c("",rownames(coe)),rbind(cnames,coe))

cor<-gm$corFixed

oo2<-cbind(c("",rownames(cor)),rbind(colnames(cor),numfmt(cor,dec)))

oo3<-rbind(c("Method:", gm$method),cbind(c("AIC:","BIC:","Log likelihood:"),numfmt(c(gm$AIC,gm$BIC,gm$logLik),dec)))

tmp<-numfmt(rbind(apply(mdl$residuals,2,mean),apply(mdl$residuals,2,sd)),4)

tmp1<-pvformat(apply(mdl$residuals,2,function(x) pearson.test(x)$p.value),4);

tmp.int<-mdl$coefficients$random$subj.ID

tmp2<-c("Random intercept", pvformat(c(mean(tmp.int),sd(tmp.int),pearson.test(tmp.int)$p.value),4))

oo4<-cbind(c("","Mean","SD","Pearson chi-square normality test"),rbind(colnames(mdl$residuals),tmp,tmp1),tmp2)

oo<-c("</br>Fixed effects<table border=3>",mat2htmltable(oo1),"</table>")

oo<-c(oo,"</br>Correlation<table border=3>",mat2htmltable(oo2),"</table>")

oo<-c(oo,"</br>Model info.<table border=3>",mat2htmltable(oo3),"</table>")

oo<-c(oo,"</br>Residuals<table border=3>",mat2htmltable(oo4),"</table>")

} else {

coe<-gm$coefficients

cnames<-c(colnames(coe),"Exp(estimate)","95%CI.low","95%CI.upp")

coe<-cbind(numfmt(coe[,(1:3)],dec),pvformat(coe[,4],4),numfmt(exp(cbind(coe[,1],coe[,1]-1.96*coe[,2],coe[,1]+1.96*coe[,2])),2))

oo1<-cbind(c("",rownames(coe)),rbind(cnames,coe))

oo2<-rbind(names(gm$AIC),numfmt(gm$AIC,dec))

oo<-c("</br>Fixed effects<table border=3>",mat2htmltable(oo1),"</table>")

oo<-c(oo,"</br>Model info.<table border=3>",mat2htmltable(oo2),"</table>")

}

} else {

print(gm)

oo<-""

}

return(oo)

}

setgamm<-function(fml,ydist) {

if (gamm==1) {

if (rdmv==0) {

if (ydist=="gaussian") mdl<-try(gamm(formula(fml),random=list(subj.ID=~1),weights=wdd$weights,data=wdd,family=gaussian))

if (ydist=="binomial") {

if (smooth==0) {

tmp.fml<-paste(fml,"+(1|subj.ID)")

mdl<-try(glmer(formula(tmp.fml),weights=wdd$weights,data=wdd,family=binomial))

} else {

mdl<-try(gamm(formula(fml),random=list(subj.ID=~1),weights=wdd$weights,data=wdd,family=binomial))

}

}

if (ydist=="poisson") mdl<-try(gamm(formula(fml),random=list(subj.ID=~1),weights=wdd$weights,data=wdd,family=poisson))

if (ydist=="gamma") mdl<-try(gamm(formula(fml),random=list(subj.ID=~1),weights=wdd$weights,data=wdd,family=Gamma))

} else {

if (rdmint==0) {

if (ydist=="gaussian") mdl<-try(gamm(formula(fml),random=list(subj.ID=~rdm.var),weights=wdd$weights,data=wdd,family=gaussian))

if (ydist=="binomial") {

tmp.fml<-paste(fml,"+(rdm.var|subj.ID)")

mdl<-try(glmer(formula(tmp.fml),weights=wdd$weights,data=wdd,family=binomial))

}

if (ydist=="poisson") mdl<-try(gamm(formula(fml),random=list(subj.ID=~rdm.var),weights=wdd$weights,data=wdd,family=poisson))

if (ydist=="gamma") mdl<-try(gamm(formula(fml),random=list(subj.ID=~rdm.var),weights=wdd$weights,data=wdd,family=Gamma))

} else {

if (ydist=="gaussian") mdl<-try(gamm(formula(fml),random=list(subj.ID=~1+rdm.var),weights=wdd$weights,data=wdd,family=gaussian))

if (ydist=="binomial") {

tmp.fml<-paste(fml,"+(1+rdm.var|subj.ID)")

mdl<-try(glmer(formula(tmp.fml),weights=wdd$weights,data=wdd,family=binomial))

}

if (ydist=="poisson") mdl<-try(gamm(formula(fml),random=list(subj.ID=~1+rdm.var),weights=wdd$weights,data=wdd,family=poisson))

if (ydist=="gamma") mdl<-try(gamm(formula(fml),random=list(subj.ID=~1+rdm.var),weights=wdd$weights,data=wdd,family=Gamma))

}

}

} else {

wdd$v <- 1/wdd$weights^2;

if (rdmv==0) {

mdl<-try(lme(formula(fml),random=list(subj.ID=~1),weights=varFixed(~v),data=wdd))

} else {

if (rdmint==0) {

mdl<-try(lme(formula(fml),random=list(subj.ID=~rdm.var),weights=varFixed(~v),data=wdd))

} else {

mdl<-try(lme(formula(fml),random=list(subj.ID=~1+rdm.var),weights=varFixed(~v),data=wdd))

}

}

}

return (mdl)

}

gamm2pngs<-function(mdl,yi,bvvstr) {

pred<-predict.gam(mdl,type="terms",se.fit=TRUE)

mfit<-NA; sfit<-NA;

cnamefit<-colnames(pred$fit)

for (k in (1:ns)) {

svnamek1<-paste("s(",svname[k],")",sep="")

svk.colmns<-which(substr(cnamefit,1,nchar(svnamek1))==svnamek1)

mfit<-cbind(mfit,apply(cbind(0,pred$fit[,svk.colmns]),1,sum));

sfit<-cbind(sfit,apply(cbind(0,pred$se.fit[,svk.colmns]),1,sum));

}

tmp.cname<-svname;

if (nx>0) {

mfit<-cbind(mfit,pred$fit[,xv1]); sfit<-cbind(sfit,pred$se.fit[,xv1]);

tmp.cname<-c(tmp.cname,xvname);

}

mfit<-matrix(mfit[,-1],ncol=length(tmp.cname)); colnames(mfit)<-paste(tmp.cname,".fit",sep="");

sfit<-matrix(sfit[,-1],ncol=length(tmp.cname)); colnames(sfit)<-paste(tmp.cname,".se",sep="");

if (mdl$family[2]=="logit") {

tmp.sh<-mean(mdl$fitted.values); tmp.sh<-log(tmp.sh/(1-tmp.sh))-apply(mfit,2,mean)

mfit<-mfit+matrix(rep(tmp.sh,nrow(mfit)),nrow=nrow(mfit),byrow=TRUE)

mfit.low<-mfit-1.96*sfit; mfit.low<-matrix(exp(mfit.low)/(1+exp(mfit.low)),ncol=length(tmp.cname))

mfit.upp<-mfit+1.96*sfit; mfit.upp<-matrix(exp(mfit.upp)/(1+exp(mfit.upp)),ncol=length(tmp.cname))

mfit<-matrix(exp(mfit)/(1+exp(mfit)),ncol=length(tmp.cname))

colnames(mfit.low)<-paste(tmp.cname,".low",sep="");

colnames(mfit.upp)<-paste(tmp.cname,".upp",sep="");

colnames(mfit)<-paste(tmp.cname,".fit",sep="");

ww<-cbind(wdd,mfit,mfit.low,mfit.upp)

} else if (mdl$family[2]=="log") {

tmp.sh<-mean(mdl$fitted.values); tmp.sh<-log(tmp.sh)-apply(mfit,2,mean)

mfit<-mfit+matrix(rep(tmp.sh,nrow(mfit)),nrow=nrow(mfit),byrow=TRUE)

mfit.low<-mfit-1.96*sfit; mfit.low<-matrix(exp(mfit.low),ncol=length(tmp.cname))

mfit.upp<-mfit+1.96*sfit; mfit.upp<-matrix(exp(mfit.upp),ncol=length(tmp.cname))

mfit<-matrix(exp(mfit),ncol=length(tmp.cname))

colnames(mfit.low)<-paste(tmp.cname,".low",sep="");

colnames(mfit.upp)<-paste(tmp.cname,".upp",sep="");

colnames(mfit)<-paste(tmp.cname,".fit",sep="");

ww<-cbind(wdd,mfit,mfit.low,mfit.upp)

} else if (mdl$family[2]=="identity") {

tmp.sh<-mean(mdl$fitted.values)-apply(mfit,2,mean)

mfit<-mfit+matrix(rep(tmp.sh,nrow(mfit)),nrow=nrow(mfit),byrow=TRUE)

ww<-cbind(wdd,mfit,sfit)

} else {

ww<-cbind(wdd,mfit,sfit)

}

xf<-paste(ofname,yvname[yi],"gamm.xls", sep="_")

if (bvvstr>"") xf<-paste(ofname,yvname[yi],bvvstr,"gamm.xls", sep="_")

write.table(ww,file=xf,row.names=FALSE,col.names=TRUE,sep="\t",append=FALSE,quote=FALSE)

px<-c(20,1:9); gg<-"<table><tr>";

for (k in (1:ns)) {

cname1<-paste(svname[k],".fit",sep=""); y.tmp<-mfit[,cname1]; y.org<-ww[,yvname[yi]]

if (mdl$family[2]=="logit" | mdl$family[2]=="log") {

cname2<-paste(svname[k],".low",sep=""); y.low<-mfit.low[,cname2]

cname3<-paste(svname[k],".upp",sep=""); y.upp<-mfit.upp[,cname3]

} else {

cname2<-paste(svname[k],".se",sep=""); se.tmp<-sfit[,cname2];

y.low<-y.tmp-1.96*se.tmp; y.upp<-y.tmp+1.96*se.tmp

}

x.tmp<-ww[,svname[k]];

pngf<-paste(ofname,yvname[yi],svname[k],"smooth.png",sep="_")

pngf0<-paste(ofname,yvname[yi],svname[k],"smooth1.png",sep="_")

if (bvvstr>"") pngf<-paste(ofname,yvname[yi],svname[k],bvvstr,"smooth.png",sep="_")

if (is.na(colvname)) {

if (chk==0) {tmp.col<-rainbow(2);} else {tmp.col<-rep("black",2);}

xy<-legLocate(c(x.tmp,x.tmp),c(y.low,y.upp))

png(pngf,width=720,height=560)

plot(y.tmp~x.tmp,ylim=c(xy[3],xy[4]),xlim=c(xy[1],xy[2]),col=tmp.col[1],type="p", pch=20, ylab="", xlab="")

par(new=TRUE);

plot(y.low~x.tmp,ylim=c(xy[3],xy[4]),xlim=c(xy[1],xy[2]),col=tmp.col[2], type="p", pch=".", ylab="", xlab="")

par(new=TRUE);

plot(y.upp~x.tmp,ylim=c(xy[3],xy[4]),xlim=c(xy[1],xy[2]),col=tmp.col[2], type="p", pch=".", ylab=yb.utf8[yi], xlab=sb.utf8[k])

dev.off()

png(pngf0,width=720,height=560)

tmp.ord<-order(x.tmp); x.tmp0<-x.tmp[tmp.ord];

y.tmp0<-y.tmp[tmp.ord];y.low0<-y.low[tmp.ord];y.upp0<-y.upp[tmp.ord]

plot(y.tmp0~x.tmp0,ylim=c(xy[3],xy[4]),xlim=c(xy[1],xy[2]),col=tmp.col[1],type="l", lty=1, lwd=2, ylab="", xlab="")

par(new=TRUE);

plot(y.low0~x.tmp0,ylim=c(xy[3],xy[4]),xlim=c(xy[1],xy[2]),col=tmp.col[2], type="l", lty=3, lwd=1, ylab="", xlab="")

par(new=TRUE);

plot(y.upp0~x.tmp0,ylim=c(xy[3],xy[4]),xlim=c(xy[1],xy[2]),col=tmp.col[2], type="l", lty=3, lwd=1, ylab=yb.utf8[yi], xlab=sb.utf8[k])

dev.off()

rm(tmp.ord,y.tmp0,x.tmp0,y.low0,y.upp0)

xy<-legLocate(x.tmp,y.org)

pngf1<-paste(ofname,yvname[yi],svname[k],"scatter.png",sep="_")

if (bvvstr>"") pngf1<-paste(ofname,yvname[yi],svname[k],bvvstr,"scatter.png",sep="_")

png(pngf1,width=720,height=560)

plot(y.tmp~x.tmp,ylim=c(xy[3],xy[4]),xlim=c(xy[1],xy[2]),col=tmp.col[1], type="p", pch=20, ylab="", xlab="")

par(new=TRUE);

plot(y.org~x.tmp,ylim=c(xy[3],xy[4]),xlim=c(xy[1],xy[2]),type="p",pch=1,cex=0.5, ylab=yb.utf8[yi], xlab=sb.utf8[k])

dev.off()

} else {

if (chk==0) {tmp.col<-rainbow(ncg);} else {tmp.col<-rep("black",ncg);}

xy<-legLocate(x.tmp,y.tmp)

png(pngf,width=720,height=560)

for (b in (1:ncg)) {

y0<-y.tmp[ww[,colvname]==colv.lv[b]]; x0<-x.tmp[ww[,colvname]==colv.lv[b]]

if (b>1) par(new=TRUE)

plot(y0~x0,ylim=c(xy[3],xy[4]),xlim=c(xy[1],xy[2]),col=tmp.col[b], type="p", pch=px[b], ylab=yb.utf8[yi], xlab=sb.utf8[k])

}

legend(xy[5],xy[6],colv.lb.utf8,pch=px[1:ncg],bty="n",col=tmp.col)

dev.off()

png(pngf0,width=720,height=560)

for (b in (1:ncg)) {

y0<-y.tmp[ww[,colvname]==colv.lv[b]]; x0<-x.tmp[ww[,colvname]==colv.lv[b]]

tmp.ord<-order(x0); x00<-x0[tmp.ord]; y00<-y0[tmp.ord];

if (b>1) par(new=TRUE)

plot(y00~x00,ylim=c(xy[3],xy[4]),xlim=c(xy[1],xy[2]),col=tmp.col[b], type="l", lty=b, lwd=2, ylab=yb.utf8[yi], xlab=sb.utf8[k])

rm(tmp.ord,x00,y00)

}

legend(xy[5],xy[6],colv.lb.utf8,lty=(1:ncg),bty="n",col=tmp.col)

dev.off()

}

gg<-c(gg,"<td>",yb[yi]," vs. ",sb[k],"</br><a href=\"",pngf,"\" target=_BLANK><img src=\"",pngf,"\" width=320,height=320></a></td>")

}

gg<-c(gg,"</tr></table>")

return(gg)

}

if (!is.na(weights.var)) {weights<-WD[,weights.var];} else {weights<-1;}

WD<-cbind(WD,weights);

TMP.ID=(1:nrow(WD)); WD.0<-cbind(TMP.ID,WD)

vlabelN<-(substr(vlabel,1,1)==" ");

vlabelZ<-vlabel[vlabelN];vlabelV<-vlabel[!vlabelN]

vnameV<-vname[!vlabelN];vnameZ<-vname[vlabelN]

xv<-cbind(WD[,xvname]); yv<-cbind(WD[,yvname])

subj.ID<-WD[,vname.start];

rdmint<-chk; nx<-0

if (!is.na(vname.stop)) {

rv<-WD[,vname.stop];

rlv<-tstoplv;

if (length(xvname)==0) {

xv<-matrix(rv,ncol=1); xvname<-vname.stop; xlv<-tstoplv;

} else if (is.na(match(vname.stop, xvname))) {

xv<-cbind(xv,rv); xvname<-c(xvname,vname.stop); xlv<-c(xlv,rlv);

}

nx<-length(xvname)

}

ny<-length(yvname);

yb<-vlabelV[match(yvname,vnameV)]; yb[is.na(yb)]<-yvname[is.na(yb)]

xb<-vlabelV[match(xvname,vnameV)]; xb[is.na(xb)]<-xvname[is.na(xb)]

yb.utf8<-yb; Encoding(yb.utf8)<-"UTF-8"

if (!is.na(bvar)) {

bv<-WD[,bvar]

bvb<-vlabelV[match(bvar,vnameV)]; if (is.na(bvb)) bvb<-bvar;

bv.lv<-levels(factor(bv));

bv.lb<-vlabelZ[match(paste(bvar,bv.lv,sep="."),vnameZ)]

bv.lb[is.na(bv.lb)]<-bv.lv[is.na(bv.lb)]

nbg<-length(bv.lv)

}

gamm0<-0; smooth<-0;

svstr<-""; nsterms<-0;

if (length(svname)>0) {

sv<-WD[,svname];

sb<-vlabelV[match(svname,vnameV)]; sb[is.na(sb)]<-svname[is.na(sb)]

sb.utf8<-sb; Encoding(sb.utf8)<-"UTF-8"

ns<-length(svname); nsterms<-ns;

sv1<-paste("s(",svname,",bs='cr'",sep="")

sdf1<-paste(",k=",sdf); sdf1[sdf==0]<-"";

sv1<-paste(sv1,sdf1)

if (!is.na(colvname)) {

colv<-WD[,colvname]

sv1<-paste(sv1,",by=factor(",colvname,")",sep="")

colv.lv<-levels(factor(colv)); ncg<-length(colv.lv);

colvb<-vlabelV[match(colvname,vnameV)]; if (is.na(colvb)) colvb<-colvname;

colv.lb<-vlabelZ[match(paste(colvname,colv.lv,sep="."),vnameZ)]

colv.lb[is.na(colv.lb)]<-colv.lv[is.na(colv.lb)]

colv.lb.utf8<-colv.lb; Encoding(colv.lb.utf8)<-"UTF-8"

nsterms<-nsterms*ncg;

}

svstr<-paste(paste(sv1,")",sep=""),collapse="+")

gamm0<-1; smooth<-1

}

xvstr<-""

if (length(xvname)>0) {

sxf<-toupper(sxf)

xv1<-xvname; xv1[xlv>2]<-paste("factor(",xv1[xlv>2],")",sep="")

xvstr<-paste(xv1,collapse="+")

} else {sxf<-c();}

if (sum(sxf=="S")>0 & !is.na(bvar)) xintrstr<-paste(paste(xv1[sxf=="S"],paste("factor(",bvar,")",sep=""),sep="*"),collapse="+")

if (svstr>"" & xvstr>"") sxstr<-paste(svstr,"+",xvstr,sep="");

if (svstr>"" & xvstr=="") sxstr<-svstr;

if (svstr=="" & xvstr>"") sxstr<-xvstr;

ofname1<-ofname;

w<-c("<!DOCTYPE html><html lang='zh'><head><meta charset='utf-8'></head><body>")

sink(paste(ofname,".lst",sep=""))

wrtWD0 <- FALSE

gam2htmltable<-function(mdl) {

gs<-summary(mdl); print(gs)

np<-length(gs$p.coeff)

coe<-rbind(NA,gs$p.table)

colp<-ncol(coe)

coep<-pvformat(coe[,colp],4)

cnames<-colnames(coe)

if (gs$family[[2]]=="log" | gs$family[[2]]=="logit") {

coe<-coe[,-colp]

cnames<-c(colnames(coe),"exp(est)","95%区间下限","95%区间上限","P.value")

coe<- cbind(coe, exp(coe[,1]), exp(coe[,1]-1.96*coe[,2]), exp(coe[,1]+1.96*coe[,2]),coep)

}

if (gs$family[[2]]=="identity") {

coe<-coe[,-colp]

cnames<-c(colnames(coe),"95%区间下限","95%区间上限","P.value")

coe<- cbind(coe, coe[,1]-1.96*coe[,2], coe[,1]+1.96*coe[,2],coep)

}

coe[,-ncol(coe)]<-numfmt(coe[,-ncol(coe)],dec)

oo1<-cbind(c("",rownames(coe[-1,])),rbind(cnames,coe[-1,]))

oo<-c("</br>线性回归项的作用<table border=3>",mat2htmltable(oo1),"</table>")

if (!is.null(gs$pTerms.table)) {

xsq<-gs$pTerms.table

xsq[,2]<-numfmt(xsq[,2],dec)

xsq[,3]<-pvformat(xsq[,3],4)

oo2<-cbind(c("",rownames(xsq)),rbind(colnames(xsq),xsq))

oo<-c(oo, "</br>线性回归项卡方检验<table border=3>",mat2htmltable(oo2),"</table>")

}

stb<-gs$s.table

stb[,c(1,2,3)]<-numfmt(stb[,c(1,2,3)],dec)

stb[,4]<-pvformat(stb[,4],4)

oo3<-cbind(c("",rownames(stb)),rbind(colnames(stb),stb))

oo<-c(oo, "</br>曲线拟合项的显著性检验<table border=3>",mat2htmltable(oo3),"</table>")

p0<-c("N:", gs$n)

p1<-c("Adj. r-square:", numfmt(gs$r.sq,4))

p2<-c("Deviance explained:", numfmt(gs$dev.expl,4))

p3<-c("Scale estimate:", numfmt(gs$scale,dec))

p4<-c("family:", gs$family[[1]])

p5<-c("link function:", gs$family[[2]])

oo4<-rbind(p0,p1,p2,p3,p4,p5)

oo<-c(oo, "</br>Model statistics<table border=3>",mat2htmltable(oo4),"</table>")

return(oo)

}

gamm2html<-function(mdl, xfname, yb1, ydist) {

yb1.utf8<-yb1; Encoding(yb1.utf8)<-"UTF-8"

oo<-paste("</br></br>结局变量:", yb1, "</br>")

if (smooth==1) {

oo<-c(oo,"</br>相加模型结果</br>")

oo<-c(oo,gam2htmltable(mdl$gam));

oo<-c(oo,"</br></br>线性混合效应</br>")

oo<-c(oo,lme2htmltable(mdl$lme));

for (g in (1:nsterms)) {

png(paste(xfname,"page",g,"smooth.png",sep="_"),width=720,height=560);

plot(mdl$gam, select=g, main=yb1.utf8)

dev.off()

}

png(paste(xfname,"gamm.png",sep="_"),width=720,height=560);

plot(mdl$lme$fitted[,"subj.ID"],mdl$lme$residuals[,"subj.ID"],xlab="Fitted value",ylab="Standardized residuals",main=yb1.utf8);

dev.off()

png(paste(xfname,"residual.png",sep="_"),width=720,height=560);

hist(mdl$lme$residuals[,"subj.ID"], plot=T, prob=T, main="残差直方图",xlab="残差")

lines(density(mdl$lme$residuals[,"subj.ID"],na.rm=TRUE))

dev.off()

} else {

oo<-paste("</br></br>线性混合效应: Y=", yb1, " distribution:", ydist, "</br>")

oo<-c(oo,lme2htmltable(mdl));

if (y.gaussian==1) {

png(paste(xfname,"lme.png",sep="_"),width=720,height=560);

plot(mdl$fitted[,"subj.ID"],mdl$residuals[,"subj.ID"],xlab="Fitted value",ylab="Standardized residuals",main=yb1.utf8);

dev.off()

png(paste(xfname,"residual.png",sep="_"),width=720,height=560);

hist(mdl$residuals[,"subj.ID"], plot=T, prob=T, main="残差直方图",xlab="残差")

lines(density(mdl$residuals[,"subj.ID"],na.rm=TRUE))

dev.off()

}

}

return(oo)

}

w<-c(w,"<h2>相加混合模型</h2>")

for (k in (1:ny)) {

yv1<-yv[,k]; yvname1<-yvname[k]; yb1<-yb[k];

rdmv<-0

yres.NAME<-paste(yvname1,c("fixed","subjID"),sep=".")

tmp.YRES<-matrix(NA,nrow=nrow(WD.0),ncol=2); colnames(tmp.YRES)<-yres.NAME

WD.0<-cbind(WD.0,tmp.YRES)

dd<-cbind(weights,TMP.ID,subj.ID,yv1); cname<-c("weights","TMP.ID","subj.ID",yvname1)

if (length(xvname)>0) {dd<-cbind(dd,xv); cname<-c(cname,xvname);}

if (length(svname)>0) {dd<-cbind(dd,sv); cname<-c(cname,svname);}

if (!is.na(bvar)) {dd<-cbind(dd,bv); cname<-c(cname,bvar);}

if (!is.na(colvname)) {dd<-cbind(dd,colv); cname<-c(cname,colvname);}

if (!is.na(vname.stop)) {cname<-c(cname,"rdm.var"); dd<-cbind(dd,rv); rdmv<-1;}

dd<-dd[apply(is.na(dd),1,sum)==0,]; colnames(dd)<-cname;

rm(WD);

WD<-as.data.frame(dd); attach(WD);

fml<-paste(yvname1,"~",sxstr,sep="")

if (!is.na(bvar)) fml0<-paste(fml,"+factor(",bvar,")",sep="")

if (sum(sxf=="S")>0 & !is.na(bvar)) fmlx<-paste(fml0,xintrstr,sep="+")

if ((ydist[k]!="gaussian") & length(svname)==0) {gamm<-1;} else {gamm<-gamm0;}

y.gaussian<-0; if (ydist[k]=="gaussian") y.gaussian<-1;

wdd<-WD;

xf<-ofname1; if (ny>1) xf<-paste(xf,yvname1,sep="_")

if (is.na(bvar)) {

tmp.mdl<-setgamm(fml,ydist[k]); print(tmp.mdl)

if (is.list(tmp.mdl)) {

errStr = substr(tmp.mdl[[1]][1],1,5);

} else {

if (is.array(tmp.mdl)) {errStr=substr(tmp.mdl[1],1,5);} else {errStr="";}

}

if (is.list(tmp.mdl)) {

if (errStr!="Error") {

w<-c(w,gamm2html(tmp.mdl, xf, yb1, ydist[k]));

if (smooth==1) w<-c(w,"</br>",gamm2pngs(tmp.mdl$gam,k,""))

if (smooth==1) {yk.resi<-tmp.mdl$lme$residuals;} else {yk.resi<-tmp.mdl$residuals;}

WD.0[match(wdd[,"TMP.ID"],WD.0[,"TMP.ID"]),yres.NAME]<-yk.resi[,c("fixed","subj.ID")]

wrtWD0<-TRUE

}

} else {if (errStr!="Error") w<-c(w,gamm2html(tmp.mdl, xf, yb1, ydist[k]));}

} else {

for (b in (1:nbg)) {

rm(wdd); wdd<-WD[(WD[,bvar]==bv.lv[b]),];

xf1<-paste(xf,bvar,bv.lv[b],sep="_")

print(xf1)

w<-c(w,paste("</br></br>亚组分析:", bvb, "=", bv.lb[b]))

tmp.mdl<-setgamm(fml,ydist[k]); print(tmp.mdl)

if (is.list(tmp.mdl)) {

errStr = substr(tmp.mdl[[1]][1],1,5);

} else {

if (is.array(tmp.mdl)) {errStr=substr(tmp.mdl[1],1,5);} else {errStr="";}

}

if (is.list(tmp.mdl)) {

if (errStr!="Error") {

w<-c(w,gamm2html(tmp.mdl, xf1, yb1, ydist[k]));

if (smooth==1) w<-c(w,"</br>",gamm2pngs(tmp.mdl$gam,k,paste(bvar,bv.lv[b],sep="")))

if (smooth==1) {yk.resi<-tmp.mdl$lme$residuals;} else {yk.resi<-tmp.mdl$residuals;}

WD.0[match(wdd[,"TMP.ID"],WD.0[,"TMP.ID"]),yres.NAME]<-yk.resi[,c("fixed","subj.ID")]

wrtWD0<-TRUE

}

} else {if (errStr!="Error") w<-c(w,gamm2html(tmp.mdl, xf1, yb1, ydist[k]));}

}

w<-c(w,paste("</br></br>合计: "))

rm(wdd); wdd<-WD; tmp.mdl<-setgamm(fml0,ydist[k]); print(tmp.mdl)

if (is.list(tmp.mdl)) {

errStr = substr(tmp.mdl[[1]][1],1,5);

} else {

if (is.array(tmp.mdl)) {errStr=substr(tmp.mdl[1],1,5);} else {errStr="";}

}

if (is.list(tmp.mdl)) {

if (errStr!="Error") w<-c(w,gamm2html(tmp.mdl, xf, yb1,ydist[k]))

} else {if (errStr!="Error") w<-c(w,gamm2html(tmp.mdl, xf, yb1,ydist[k]));}

if (sum(sxf=="S")>0) {

w<-c(w,paste("</br></br>分组因素与选择的自变量交互作用分析：", bvb, "with", paste(xb[sxf=="S"], collapse=" ")))

rm(wdd); wdd<-WD; tmp.mdl<-setgamm(fmlx,ydist[k]);

if (is.list(tmp.mdl)) {

errStr = substr(tmp.mdl[[1]][1],1,5);

} else {

if (is.array(tmp.mdl)) {errStr=substr(tmp.mdl[1],1,5);} else {errStr="";}

}

if (is.list(tmp.mdl)) {

if (errStr!="Error") w<-c(w,gamm2html(tmp.mdl, xf, yb1, ydist[k]))

} else {if (errStr!="Error") w<-c(w,gamm2html(tmp.mdl, xf, yb1, ydist[k]));}

}

}

}

sink()

w<-c(w,wd.subset)

w<-c(w,paste("</br></br>Created by EmpowerStats (www.empowerstats.com) and R on",Sys.Date()))

w<-c(w,"</body></html>")

fileConn<-file(paste(ofname,".htm",sep="")); writeLines(w, fileConn)

if (wrtWD0) write.table(WD.0,file=paste(ofname,"_residuals.xls",sep=""),row.names=FALSE,col.names=TRUE,sep="\t")
